# Supplementary material for: Functioning of People with Lipoedema According to All Domains of the International Classification of Functioning, Disability and Health: A Scoping Review
Source: Int J Environ Res Public Health. 2023 Jan 21;20(3):1989. doi: 10.3390/ijerph20031989 (PMC9915552; doi:10.3390/ijerph20031989)
Supplement: Supplementary file 1 [file ijerph-20-01989-s001.zip › Supplementary files A L.M. Kloosterman.pdf]

Supplementary Table S1. Search strings

Search string Pubmed

("Lipedema"[MeSH] OR liped*[tiab] OR lipoed*[tiab] OR Lipolymph*[tiab]) AND ("Nutritional Status"[Mesh] OR nutri*[tiab] OR "Malnutrition"[Mesh] OR malnutrition[tiab] OR undernutrition[tiab] OR malnourish*[tiab] OR undernourish*[tiab] OR "Avitaminosis"[Mesh] OR avitaminosis[tiab] OR "Deficiency Diseases"[Mesh] OR deficiency dis*[tiab] OR "Protein Deficiency"[Mesh] OR protein deficienc*[tiab] OR protein-energy malnutrition[tiab] OR "Overnutrition"[Mesh] OR overnutrition[tiab] OR "Hypervitaminosis A"[Mesh] OR hypervitaminosis A[tiab] OR "Feeding and Eating Disorders"[Mesh] OR feeding dis*[tiab] OR eating dis*[tiab] OR "Binge-Eating Disorder"[Mesh] OR binge-eating dis*[tiab] OR physical funct*[tiab] OR "Physical Fitness"[Mesh] OR physical fitness[tiab] OR "Cardiorespiratory Fitness"[Mesh] OR cardiorespiratory*[tiab] OR physical abilit*[tiab] OR physical health[tiab] OR functional abilit*[tiab] OR "Exercise"[Mesh] OR exercise*[tiab] OR physical activit*[tiab] OR rand 36[tiab] OR SF 36[tiab] OR 36 Item Short Form Health Survey[tiab] OR Short Form*[tiab] OR EQ-5D*[tiab] OR EuroQuol 5D[tiab] OR Musculoskeletal Function Assessment[tiab] OR MFA[tiab] OR SMFA[tiab] OR WHOQOL*[tiab] OR Lower Extremity Functional Scale[tiab] OR LEFS[tiab] OR Short Questionnaire to Assess Health-enhancing physical activity[tiab] OR SQUASH[tiab] OR LASA Physical Activity Questionnaire[tiab] OR LAPAQ[tiab] OR Pedometer[tiab] OR Patient Specific Complaints List[tiab] OR PSK[tiab] OR Multidimensional Fatigue Index[tiab] OR MFI[tiab] OR MVI[tiab] OR MFI-20[tiab] OR Central Sensitization Inventory[tiab] OR CSI[tiab] OR 6MWT[tiab] OR walk test*[tiab] OR walk* distance[tiab] OR 6MWD[tiab] OR steep ramp test[tiab] OR SRT[tiab] OR "Quadriceps Muscle"[Mesh] OR Quadriceps Muscle*[tiab] OR Quadriceps Femoris[tiab] OR sit to stand[tiab] OR Timed Chair Stand Test[tiab] OR TCST[tiab] OR FTSTS[tiab] OR 10MWT[tiab] OR "Exercise Test"[Mesh] OR "Hand Strength"[Mesh] OR hand* strength[tiab] OR handgrip[tiab] OR grip strength[tiab] OR hand held dynamometer[tiab] OR graps strength[tiab] OR "Depression"[Mesh] OR depress*[tiab] OR stress[tiab] OR psychological functioning[tiab] OR kinesiophobia[tiab] OR fear of movement[tiab] OR "fear"[Mesh] OR fear*[tiab] OR “Psychosocial Functioning”[Mesh] OR Psychosocial Function*[tiab] OR "Panic"[Mesh] OR panic[tiab] OR "Mental Health"[Mesh] OR mental health[tiab] OR Psych*[tiab] OR mental[tiab] OR "Pain"[Mesh] OR pain*[tiab] OR "Acute Pain"[Mesh] OR acute pain[tiab] OR "Breakthrough Pain"[Mesh] breakthrough pain[tiab] OR "Musculoskeletal Pain"[Mesh] OR Musculoskeletal pain[tiab] OR "Chronic Pain"[Mesh] OR chronic pain[tiab] OR "Nociceptive Pain"[Mesh] OR nociceptive pain[tiab] OR "Pain Perception"[Mesh] OR pain perception[tiab] OR "Pain, Referred"[Mesh] OR Physical Suffering*[tiab] OR Ache*[tiab] OR Visual Analogue Scale[tiab] OR VAS[tiab] OR numeric pain rating scale[tiab] OR numeric rating scale[tiab] OR NPRS[tiab] OR NRS[tiab] OR Muscle fat*[tiab] OR muscle-to-fat*[tiab] OR muscle/fat*[tiab] OR fat-muscle[tiab] OR fat/muscle*[tiab] OR fat-to-muscle*[tiab] OR fat-muscle*[tiab] OR fat ratio[tiab] OR muscle ratio[tiab] OR body composition[tiab] OR "Body Composition"[Mesh] OR muscle tissue distribution[tiab] OR adipose tissue*[tiab] OR "Muscles"[Mesh] OR "Sarcopenia"[Mesh] OR "Adiposity"[Mesh] OR Muscle*[tiab] OR Sarcopenia[tiab] OR Adiposity[tiab] OR Lean mass[tiab] OR Fat-free*[tiab] OR Fat mass*[tiab] OR "Magnetic Resonance Imaging"[Mesh] OR "Ultrasonography"[Mesh] OR "Absorptiometry, Photon"[Mesh] OR Magnetic Resonance Imaging[tiab] OR Ultrasonography[tiab] OR ultrasound[tiab] OR dual energy X-ray absorptiometry[tiab] OR echography[tiab] OR BIA[tiab] OR bio electrical impendance analysis[tiab] OR bio impendance*[tiab] OR “Obesity”[Mesh] OR obesity[tiab] OR obese[tiab] OR "Obesity Hypoventilation Syndrome"[Mesh] OR "Obesity, Abdominal"[Mesh] OR "Obesity, Maternal"[Mesh] OR "Obesity, Metabolically Benign"[Mesh] OR "Obesity, Morbid"[Mesh] OR "Pediatric Obesity"[Mesh] OR fatigue severity scale[tiab] OR FSS[tiab] OR fatigue*[tiab] OR "Fatigue"[Mesh] OR Shortened fatigue questionnaire[tiab] OR SFQ[tiab] OR FAS[tiab] OR multidimensional assessment of fatigue scale[tiab] OR MAF[tiab] OR tiredness[tiab] OR "Fatigue Syndrome, Chronic"[Mesh] OR "International Classification of Functioning, Disability and Health"[Mesh] OR "Psychosocial Functioning"[Mesh] OR "Social Interaction"[Mesh] OR International Classification of Functioning, Disability and Health[tiab] OR Social Interaction[tiab] OR "Cognition"[Mesh] OR "Sensory Gating"[Mesh] OR "Voice"[Mesh] OR "Respiratory Physiological

Phenomena"[Mesh] OR "Immune System Diseases"[Mesh] OR "Cardiovascular System"[Mesh] OR "Endocrine System"[Mesh] OR "Digestive System"[Mesh] OR "Genitalia"[Mesh] OR "Movement"[Mesh] OR "Skin Physiological Phenomena"[Mesh] OR "Nervous System"[Mesh] OR "Eye"[Mesh] OR "Learning"[Mesh] OR "Knowledge"[Mesh] OR "Communication"[Mesh] OR "Mobility Limitation"[Mesh] OR "Social Mobility"[Mesh] OR "Range of Motion, Articular"[Mesh] OR "Self Care"[Mesh] OR "Household Work"[Mesh] OR "Empathy"[Mesh] OR "Interpersonal Relations"[Mesh] OR "Education"[Mesh] OR "Work"[Mesh] OR "Employment"[Mesh] OR "Environment"[Mesh] OR "Activities of Daily Living"[Mesh] OR "Social Participation"[Mesh] OR "Community Participation"[Mesh] OR "Work Engagement"[Mesh] OR "Social Support"[Mesh] OR "Family"[Mesh] OR "Attitude"[Mesh] OR "Attitude to Health"[Mesh] OR "Health Knowledge, Attitudes, Practice"[Mesh] OR Cognition[tiab] OR Sensory Gating[tiab] OR Voice[tiab] OR Respiratory Physiological Phenomena[tiab] OR Immune System Diseases[tiab] OR Cardiovascular System[tiab] OR Endocrine System[tiab] OR Digestive System[tiab] OR Genitalia[tiab] OR Movement[tiab] OR Skin Physiological Phenomena[tiab] OR Nervous System[tiab] OR Eye[tiab] OR Learning[tiab] OR Knowledge[tiab] OR Communication[tiab] OR Mobility Limitation[tiab] OR Social Mobility[tiab] OR Range of Motion[tiab] OR Self Care[tiab] OR Household Work[tiab] OR Empathy[tiab] OR Interpersonal Relations[tiab] OR Education[tiab] OR Work[tiab] OR Employment[tiab] OR Environment[tiab] OR Activities of Daily Living[tiab] OR ADL[tiab] OR Social Participation[tiab] OR Community Participation[tiab] OR Work Engagement[tiab] OR Social Support[tiab] OR Family[tiab] OR Attitude*[tiab] OR activit*[tiab] OR participation[tiab] OR body functions[tiab] OR body function[tiab] OR body systems[tiab] OR body system[tiab] OR habits[tiab] OR coping[tiab] OR lifestyle[tiab] OR social status[tiab])

Search string Cinahl

(TI ((Lipolymph* OR liped* OR lipoed*)) OR AB ((Lipolymph* OR liped* OR lipoed*)) OR (MM Lipedema)) AND

(TI ((Nutri* OR malnutrition OR Nutritional Deficienc* OR Undernutrition OR Malnourish* OR Avitaminosis OR Deficiency Dis* OR Protein Deficienc* OR Protein-Energy Malnutrition OR Overnutrition OR Hypervitaminosis A OR Feeding and Eating Dis* OR Binge-Eating Dis* OR eating dis* OR undernutrition OR Undernourish*)) OR AB ((Nutri* OR malnutrition OR Nutritional Deficienc* OR Undernutrition OR Malnourish* OR Avitaminosis OR Deficiency Dis* OR Protein Deficienc* OR Protein-Energy Malnutrition OR Overnutrition OR Hypervitaminosis A OR Feeding and Eating Dis* OR Binge-Eating Dis* OR eating dis* OR undernutrition OR Undernourish*)) OR (((MM "Protein-Energy Malnutrition" OR "eating disorder" OR "undernutrition" OR "Undernourishment" OR "Undernourished" OR "Malnourished") OR (MM "Nutritional Status") OR (MM "Nutritional Status: Energy (Iowa NOC)") OR (MM "Nutritional Status: Food & Fluid Intake (Iowa NOC)") OR (MM "Nutritional Status: Nutrient Intake (Iowa NOC)") OR (MM "Eating Disorders") OR (MM "Binge Eating Disorder") OR (MM "Bulimia Nervosa") OR (MM "Malnutrition")))) OR TI ((physical funct* OR physical fitness OR cardiorespiratory* OR physical abilit* OR physical health OR functional abilit* OR exercise* OR physical activit*)) OR AB ((physical funct* OR physical fitness OR cardiorespiratory* OR physical abilit* OR physical health OR functional abilit* OR exercise* OR physical activit*)) OR (((MM "Physical Examination") OR (MM "Cardiorespiratory Fitness") OR (MM "Physical Fitness") OR (MM "Health Status") OR (MM "Functional Status") OR (MM "Functional Assessment") OR (MM "Exercise Test, Muscular") OR (MM "Exercise Test") OR (MM "Exercise Test, Cardiopulmonary") OR (MM "Athletic Ability") OR (MM "Health Beliefs: Perceived Ability to Perform (Iowa NOC)") OR (MM "Physical Mobility") OR (MM "Physical Endurance")))) OR TI ((rand 36 OR SF 36 OR 36 Item Short Form Health Survey OR Short Form* OR EQ-5D* OR EuroQuol 5D OR Musculoskeletal Function Assessment OR MFA OR SMFA OR WHOQOL* OR Lower Extremity Functional Scale OR LEFS OR Short Questionnaire to Assess Health-enhancing physical activity OR SQUASH OR LASA Physical Activity Questionnaire OR LAPAQ OR Pedometer OR Patient Specific Complaints List OR PSK OR Multidimensional Fatigue Index OR MFI OR MVI OR MFI-20 OR Central Sensitization Inventory OR CSI)) OR AB ((rand 36 OR SF 36 OR 36 Item Short Form Health Survey

OR Short Form* OR EQ-5D* OR EuroQuol 5D OR Musculoskeletal Function Assessment OR MFA OR SMFA OR WHOQOL* OR Lower Extremity Functional Scale OR LEFS OR Short Questionnaire to Assess Health-enhancing physical activity OR SQUASH OR LASA Physical Activity Questionnaire OR LAPAQ OR Pedometer OR Patient Specific Complaints List OR PSK OR Multidimensional Fatigue Index OR MFI OR MVI OR MFI-20 OR Central Sensitization Inventory OR CSI)) OR (MM "Short Form-36 Health Survey (SF-36)")) OR TI ((6MWT OR walk test* OR walk* distance OR 6MWD OR steep ramp test OR SRT OR Quadriceps Muscle* OR Quadriceps Femoris OR sit to stand OR Timed Chair Stand Test OR TCST OR FTSTS OR 10MWT OR hand* strength OR handgrip OR grip strength OR hand held dynamometer OR graps strength)) OR AB ((6MWT OR walk test* OR walk* distance OR 6MWD OR steep ramp test OR SRT OR Quadriceps Muscle* OR Quadriceps Femoris OR sit to stand OR Timed Chair Stand Test OR TCST OR FTSTS OR 10MWT OR hand* strength OR handgrip OR grip strength OR hand held dynamometer OR graps strength)) OR ((MM "Quadriceps Muscles") OR (MM "Grip Strength") OR (MM "Muscle Strength") OR (MM "Exercise Test, Muscular"))) OR TI ((depress* OR stress OR psychological functioning OR kinesiophobia OR fear of movement OR fear* OR Psychosocial Function* OR panic OR mental health OR Psych* OR mental)) OR AB ((depress* OR stress OR psychological functioning OR kinesiophobia OR fear of movement OR fear* OR Psychosocial Function* OR panic OR mental health OR Psych* OR mental)) OR (((MM "Depression") OR (MM "Kinesiophobia") OR (MM "Fear") OR (MM "Psychosocial Aspects of Illness") OR (MM "Psychological Tests") OR (MM "Rehabilitation, Psychosocial") OR (MM "Psychosocial Functioning") OR (MM "Psychosocial Adjustment: Life Change (Iowa NOC)") OR (MM "Stress") OR (MM "Stress, Psychological")))) OR TI ((pain* OR acute pain OR breakthrough pain OR Musculoskeletal pain OR chronic pain OR nociceptive pain OR pain perception OR Physical Suffering* OR Ache* OR Visual Analogue Scale OR VAS OR numeric pain rating scale OR numeric rating scale OR NPRS OR NRS)) OR AB ((pain* OR acute pain OR breakthrough pain OR Musculoskeletal pain OR chronic pain OR nociceptive pain OR pain perception OR Physical Suffering* OR Ache* OR Visual Analogue Scale OR VAS OR numeric pain rating scale OR numeric rating scale OR NPRS OR NRS)) OR (((MM "Pain") OR (MM "Nociceptive Pain") OR (MM "Pain Measurement") OR (MM "Chronic Pain") OR (MM "Pain Management") OR (MM "Myofascial Pain Syndromes") OR (MM "Visual Analog Scaling")))) OR TI ((Muscle fat* OR muscle-to-fat* OR muscle/fat* OR fat-muscle OR fat/muscle* OR fat-to-muscle* OR fat-muscle* OR fat ratio OR muscle ratio OR body composition OR muscle tissue distribution OR adipose tissue* OR Muscle* OR Sarcopenia OR Adiposity OR Lean mass OR Fat-free* OR Fat mass* OR Magnetic Resonance Imaging OR Ultrasonography OR ultrasound OR dual energy X-ray absorptiometry OR echography OR BIA OR bio electrical impendance analysis OR bio impendance* OR obesity OR obese)) OR AB ((Muscle fat* OR muscle-to-fat* OR muscle/fat* OR fat-muscle OR fat/muscle* OR fat-to-muscle* OR fat-muscle* OR fat ratio OR muscle ratio OR body composition OR muscle tissue distribution OR adipose tissue* OR Muscle* OR Sarcopenia OR Adiposity OR Lean mass OR Fat-free* OR Fat mass* OR Magnetic Resonance Imaging OR Ultrasonography OR ultrasound OR dual energy X-ray absorptiometry OR echography OR BIA OR bio electrical impendance analysis OR bio impendance* OR obesity OR obese)) OR (((MM "Body Composition") OR (MM "Fat Free Mass") OR (MM "Adipose Tissue Distribution") OR (MM "Magnetic Resonance Imaging") OR (MM "Ultrasonography") OR (MM "Ultrasound Technologists") OR (MM "Electric Impedance")))) OR (TI ((Obesity OR "Obesity Hypoventilation Syndrome" OR "Obesity, Abdominal" OR "Obesity, Maternal" OR "Obesity, Metabolically Benign" OR "Obesity, Morbid" OR "Pediatric Obesity" OR "Abdominal Obesities" OR "Obesities, Abdominal" OR "Abdominal Obesity" OR "Central Obesity" OR "Central Obesities" OR "Obesities, Central" OR "Obesity, Central" OR "Obesity, Visceral" OR "Visceral Obesity" OR "Obesities, Visceral" OR "Visceral Obesities" OR "obese")) OR AB ((Obesity OR "Obesity Hypoventilation Syndrome" OR "Obesity, Abdominal" OR "Obesity, Maternal" OR "Obesity, Metabolically Benign" OR "Obesity, Morbid" OR "Pediatric Obesity" OR "Abdominal Obesities" OR "Obesities, Abdominal" OR "Abdominal Obesity" OR "Central Obesity" OR "Central Obesities" OR "Obesities, Central" OR "Obesity, Central" OR "Obesity, Visceral" OR "Visceral Obesity" OR "Obesities, Visceral" OR "Visceral Obesities" OR "obese")) OR (((MM "Obesity") OR (MM "Obesity, Morbid"))))

OR TI ((fatigue severity scale OR FSS OR fatigue* OR Shortened fatigue questionnaire OR SFQ OR FAS OR multidimensional assessment of fatigue scale OR MAF OR tiredness)) OR AB ((fatigue severity scale OR FSS OR fatigue* OR Shortened fatigue questionnaire OR SFQ OR FAS OR multidimensional assessment of fatigue scale OR MAF OR tiredness)) OR (((MM "Fatigue") OR (MM "Fatigue Syndrome, Chronic") OR (MM "Muscle Fatigue")))) OR ((MH "Cognition+") OR (MH "Sensation+") OR (MM "International Classification of Functioning, Disability, and Health") OR (MH "Voice+") OR (MH "Respiratory System+") OR (MH "Immune System+") OR (MH "Cardiovascular System+") OR (MH "Endocrine System+") OR (MH "Digestive System+")OR (MH "Genitalia+") OR (MH "Movement+") OR (MH "Skin+") OR (MH "Nervous System+") OR (MH "Eye+") OR (MH "Learning+") OR (MH "Knowledge+") OR (MH "Communication+") OR (MM "Physical Mobility") OR (MM "Social Mobility") OR (MH "Mobility (Iowa NOC)+") OR (MH "Self Care+") OR (MM "Empathy") OR (MH "Interpersonal Relations+") OR (MH "Education+") OR (MH "Work+") OR (MH "Environment+") OR (MH "Employment+") OR (MH "Activities of Daily Living+") OR (MM "Social Participation") OR (MM "Work Engagement") OR (MH "Support, Psychosocial+") OR (MM "Decision Support Systems, Clinical") OR (MM "Decision Support Systems, Management") OR "support" OR (MH "Emotional Support (Saba CCC)+") OR (MH "Family+") OR (MH "Attitude+") OR (MH "Attitude to Illness+") OR (MH "Attitude of Health Personnel+") OR (MM "Attitude to Mental Illness")) OR TI (International Classification of Functioning, Disability and Health OR Social Interaction OR Cognition OR Sensory Gating OR Voice OR Respiratory Physiological Phenomena OR Immune System Diseases OR Cardiovascular System OR Endocrine System OR Digestive System OR Genitalia OR Movement OR Skin Physiological Phenomena OR Nervous System OR Eye OR Learning OR Knowledge OR Communication OR Mobility Limitation OR Social Mobility OR Range of Motion OR Self Care OR Household Work OR Empathy OR Interpersonal Relations OR Education OR Work OR Employment OR Environment OR Activities of Daily Living OR ADL OR Social Participation OR Community Participation OR Work Engagement OR Social Support OR Family OR Attitude* OR activit* OR participation OR body functions OR body function OR body systems OR body system OR habits OR coping OR lifestyle OR social status)) OR AB (International Classification of Functioning, Disability and Health OR Social Interaction OR Cognition OR Sensory Gating OR Voice OR Respiratory Physiological Phenomena OR Immune System Diseases OR Cardiovascular System OR Endocrine System OR Digestive System OR Genitalia OR Movement OR Skin Physiological Phenomena OR Nervous System OR Eye OR Learning OR Knowledge OR Communication OR Mobility Limitation OR Social Mobility OR Range of Motion OR Self Care OR Household Work OR Empathy OR Interpersonal Relations OR Education OR Work OR Employment OR Environment OR Activities of Daily Living OR ADL OR Social Participation OR Community Participation OR Work Engagement OR Social Support OR Family OR Attitude* OR activit* OR participation OR body functions OR body function OR body systems OR body system OR habits OR coping OR lifestyle OR social status))

Search string Cochrane

("Lipedema":ti,ab OR "Lipedemas":ti,ab OR "Lipoedema":ti,ab OR "Lipolymphedema":ti,ab OR "liped*":ti,ab OR "lipoed*":ti,ab OR [mh ^"lipedema"]) AND ("nutri*":ti,ab OR "malnutr*":ti,ab OR "Nutritional Deficienc*":ti,ab OR "Undernutri*":ti,ab OR "Malnourish*":ti,ab OR "Avitaminosis":ti,ab OR "Deficiency Diseas*":ti,ab OR "Protein Deficienc*":ti,ab OR "Protein-Energy Malnutrition":ti,ab OR "Overnutri*":ti,ab OR "Hypervitaminosis A":ti,ab OR "Feeding and Eating Dis*":ti,ab OR "Binge-Eating Diso*":ti,ab OR [mh "Feeding and Eating Disorders"] OR [mh "nutrition assessment"] OR [mh "nutrition disorders"] OR [mh "malnutrition"] OR "physical funct*":ti,ab OR "physical Fitness":ti,ab OR "Cardiorespiratory Fitness":ti,ab OR "physical ability*":ti,ab OR "physical health":ti,ab OR "functional ability*":ti,ab OR "Exercise*":ti,ab OR "Physical Activit*":ti,ab OR [mh ^" Health Status"] OR [mh "Funtional status"] OR [mh "Exercise Test"] OR [mh "Cardiorespiratory Fitness"] OR [mh "physical fitness"] OR "rand 36":ti,ab OR "SF 36":ti,ab OR "36 Item Short Form Health Survey":ti,ab OR "Short Form*":ti,ab OR "EQ 5D*":ti,ab OR "EuroQuol 5D":ti,ab OR "Musculoskeletal Function Assessment":ti,ab OR "MFA":ti,ab OR "SMFA":ti,ab OR "WHOQOL*":ti,ab OR "Lower Extremity Functional Scale":ti,ab OR "LEFS":ti,ab OR "Short Questionnaire to Assess Health-enhancing physical

activity":ti,ab OR "SQUASH":ti,ab OR "LASA Physical Activity Questionnaire":ti,ab OR "LAPAQ":ti,ab OR "Pedometer":ti,ab OR "Patient Specific Complaints List":ti,ab OR "PSK":ti,ab OR "Multidimensional Fatigue Index":ti,ab OR "MFI":ti,ab OR "MVI":ti,ab OR "MVI 20":ti,ab OR "MFI 20":ti,ab OR "Central Sensitization Inventory":ti,ab OR "CSI":ti,ab OR "6MWT":ti,ab OR "walk test":ti,ab OR "6MWD":ti,ab OR "walk* distance":ti,ab OR "steep ramp test":ti,ab OR SRT:ti,ab OR "Quadriceps Muscle*":ti,ab OR "Quadriceps Femoris":ti,ab OR "Timed Chair Stand Test":ti,ab OR "sit to Stand":ti,ab OR "TCST":ti,ab OR "FTSTS":ti,ab OR "FRSTS":ti,ab OR "10MWT":ti,ab OR "Exercise Test":ti,ab OR "Hand Strength":ti,ab OR "handgrip":ti,ab OR "grip strength":ti,ab OR "Handgrip strength":ti,ab OR "hand held dynamometer":ti,ab OR "graps strength":ti,ab OR [mh "Walk Test"] OR [mh "Quadriceps Muscle"] OR [mh "muscle, skeletal"] OR "depress*":ti,ab OR "stress":ti,ab OR "psychological funct*":ti,ab OR "kinesiophobia":ti,ab OR "fear*":ti,ab OR "Psychosocial Funct*":ti,ab OR "panic":ti,ab OR "mental health":ti,ab OR "Psych*":ti,ab OR "mental":ti,ab OR [mh "Depression"] OR [mh ^"Fear"] OR [mh "Psychosocial Functioning"] OR [mh "Anxiety Disorders"] OR "Pain*":ti,ab OR "*pain":ti,ab OR "Physical Suffering*":ti,ab OR "Ache*":ti,ab OR "Visual Analogue Scale":ti,ab OR "VAS":ti,ab OR "numeric pain rating scale":ti,ab OR "numeric rating scale":ti,ab OR "NPRS":ti,ab OR "NRS":ti,ab OR [mh "Pain"] OR "Muscle-fat*":ti,ab OR "muscle-to-fat*":ti,ab OR "fat-muscle*":ti,ab OR "fat-to-muscle*":ti,ab OR "fat ratio":ti,ab OR "muscle ratio":ti,ab OR "body composition":ti,ab OR "muscle tissue*":ti,ab OR "tissue distribution":ti,ab OR "adipose tissue*":ti,ab OR "Magnetic Resonance Imaging":ti,ab OR "Ultrasonography":ti,ab OR "Absorptiometry, Photon":ti,ab OR "ultrasound":ti,ab OR "dual energy X-ray absorptiometry":ti,ab OR "echography":ti,ab OR "BIA":ti,ab OR "bio electrical impendance analysis":ti,ab OR "bio impendance analysis":ti,ab OR [mh "Ultrasonography"] OR [mh "Diagnostic Imaging"] OR [mh "Magnetic Resonance Angiography"] OR [mh "Electric Impedance"] OR [mh "Body Composition"] OR [mh "body fat distribution"] OR [mh "muscles"] OR [mh "sarcopenia"] OR [mh "adiposity"] OR Muscle*:ti,ab OR "Lean mass":ti,ab OR "Fat-free mass":ti,ab OR "Fat mass*":ti,ab OR "sarcopenia":ti,ab OR "adiposity":ti,ab OR Obes*:ti,ab OR [mh "obesity"] OR "fatigue*":ti,ab OR "FSS":ti,ab OR "SFQ":ti,ab OR "FAS":ti,ab OR "multidimensional assessment of fatigue scale":ti,ab OR "MAF":ti,ab OR "tiredness":ti,ab OR [mh "Fatigue"] OR [mh "International Classification of Functioning Disability and Health"] OR "International Classification of Functioning Disability and Health":ti,ab OR "ICF":ti,ab OR [mh "cognition"] OR "Cognition":ti,ab OR [mh "sensation"] OR "sensation":ti,ab OR [mh "voice"] OR "voice":ti,ab OR [mh "Respiratory system"] OR "Respiratory system":ti,ab OR [mh "immune system"] OR "immune system":ti,ab OR [mh "cardiovascular system"] OR "cardiovascular system":ti,ab OR [mh "Endocrine System"] OR "endocrine system":ti,ab OR [mh "Digestive system"] OR "digestive system":ti,ab OR [mh "genitalia"] OR "genitalia":ti,ab OR [mh "movement"] OR "movement":ti,ab OR [mh "skin"] OR "skin":ti,ab OR [mh "nervous system"] OR "nervous system":ti,ab OR [mh "eye"] OR "eye":ti,ab OR [mh "learning"] OR "learning":ti,ab OR [mh "knowledge"] OR "knowledge":ti,ab OR [mh "communication"] OR "communication":ti,ab OR [mh "mobility limitation"] OR "mobility":ti,ab OR [mh "social mobility"] OR "social mobility":ti,ab OR [mh "self care"] OR "self care":ti,ab OR [mh "household"] OR "household":ti,ab OR [mh "empathy"] OR "empathy":ti,ab OR [mh "interpersonal relation"] OR "interpersonal relation":ti,ab OR [mh "education"] OR "education":ti,ab OR [mh "work"] OR "work":ti,ab OR [mh "employment"] OR "employment":ti,ab OR [mh "environment"] OR "environment":ti,ab OR [mh "activities of daily living"] OR "activities of daily living":ti,ab OR [mh "social participation"] OR "participation":ti,ab OR [mh "patient participation"] OR "patient participation":ti,ab OR [mh "work engagement"] OR "work engagement":ti,ab OR [mh "social support"] OR "social support":ti,ab OR [mh "family"] OR "family":ti,ab OR [mh "attitude"] OR "attitude":ti,ab OR "body function":ti,ab OR "body functions":ti,ab OR "body system":ti,ab OR "body systems":ti,ab OR "International Classification of Functioning Disability and Health":ti,ab OR "Psychosocial Functioning":ti,ab OR "Social Interaction":ti,ab OR "International Classification of Functioning Disability and Health":ti,ab OR "Psychosocial Functioning":ti,ab OR "Social Interaction":ti,ab OR [mh "social interaction"] OR "habits":ti,ab OR "coping":ti,ab OR "lifestyle":ti,ab OR "social status":ti,ab OR [mh "habits"] OR [mh "lifestyle"])

Search string Embase

('lipedema'/exp OR 'liped*':ab,ti OR 'lipoed*':ab,ti OR 'lipolymphedema':ab,ti) AND ('nutrition'/exp OR 'Nutriti*':ab,ti OR 'Malnutrition'/exp OR 'malnutrition':ab,ti OR 'Nutritional Deficienc*':ab,ti OR 'Undernutrition':ab,ti OR 'Malnourish*':ab,ti OR 'avitaminosis':ab,ti OR 'Deficiency Dis*':ab,ti OR 'Protein Deficienc*':ab,ti OR 'Protein-Energy Malnutrition':ab,ti OR 'protein calorie malnutrition'/exp OR 'Overnutrition':ab,ti OR 'Hypervitaminosis A':ab,ti OR 'protein deficiency'/exp OR 'protein deficienc*':ab,ti OR 'eating dis*':ab,ti OR 'eating dis*':ab,ti OR 'undernourish*':ab,ti OR 'physical funct*':ab,ti OR 'physical ability*':ab,ti OR 'physical health':ab,ti OR 'functional abilit*':ab,ti OR 'exercise*':ab,ti OR 'physical activit*':ab,ti OR 'physical fitness':ab,ti OR 'cardiorespiratory*':ab,ti OR 'physical performance'/exp OR 'physical performance':ab,ti OR 'rand 36':ab,ti OR 'sf 36':ab,ti OR '36 item short form health survey':ab,ti OR 'short form*':ab,ti OR 'eq-5d*':ab,ti OR 'euroquol 5d':ab,ti OR 'musculoskeletal function assessment':ab,ti OR 'mfa':ab,ti OR 'smfa':ab,ti OR 'whoqol*':ab,ti OR 'lower extremity functional scale':ab,ti OR 'lefs':ab,ti OR 'short questionnaire to assess health-enhancing physical activity':ab,ti OR 'squash':ab,ti OR 'lasa physical activity questionnaire':ab,ti OR 'lapaq':ab,ti OR 'pedometer':ab,ti OR 'patient specific complaints list':ab,ti OR 'psk':ab,ti OR 'multidimensional fatigue index':ab,ti OR 'mfi*':ab,ti OR 'mvi*':ab,ti OR 'central sensitization inventory':ab,ti OR 'csi':ab,ti OR 6mwt:ab,ti OR 'walk test*':ab,ti OR '6mwd':ab,ti OR 'walk* distance':ab,ti OR 'steep ramp test':ab,ti OR 'SRT':ab,ti OR 'quadriceps muscle*':ab,ti OR 'quadriceps femoris':ab,ti OR 'sit tot stand':ab,ti OR 'timed chair stand test':ab,ti OR 'tcst':ab,ti OR 'ftsts':ab,ti OR 'frsts':ab,ti OR '10mwt':ab,ti OR 'exercise test':ab,ti OR 'hand strength':ab,ti OR 'hand* strength':ab,ti OR 'handgrip':ab,ti OR 'grip strength*':ab,ti OR 'hand held dynamometer':ab,ti OR 'grasp strength':ab,ti OR 'grip strength test'/exp OR 'depress*':ab,ti OR 'depression'/exp OR 'stress':ab,ti OR 'psychological funct*':ab,ti OR 'kinesiophobia':ab,ti OR 'kinesiophobia'/exp OR 'fear*':ab,ti OR 'fear'/exp OR 'psychosocial funct*':ab,ti OR 'panic':ab,ti OR 'behavior'/exp OR 'behavior':ab,ti OR 'mental disease'/exp OR 'mental diseas*':ab,ti OR 'social psychology'/exp OR 'social psychology':ab,ti OR 'mental function'/exp OR 'mental funct*':ab,ti OR "mental health":ab,ti OR "Psych*":ab,ti OR "mental":ab,ti OR Pain*:ab,ti OR 'pain'/exp OR 'musculoskeletal pain'/exp OR 'nociception'/exp OR 'nociception':ab,ti OR 'pain assessment'/exp OR 'physical suffering':ab,ti OR 'ache*':ab,ti OR 'Visual Analogue Scale':ab,ti OR 'VAS':ab,it OR 'numeric pain rating scale':ab,ti OR 'numeric rating scale':ab,ti OR 'NPRS':ab,ti OR 'NRS':ab,ti OR 'Muscle-fat*':ab,ti OR 'muscle-to-fat*':ab,ti OR 'fat-muscle*':ab,ti OR 'fat-to-muscle':ab,ti OR 'fat-muscle*':ab,ti OR 'fat ratio':ab,ti OR 'body composition':ab,ti OR 'body composition analyzer'/exp OR 'muscle tissue*':ab,ti OR 'tissue distribution':ab,ti OR 'adipose tissue*':ab,ti OR 'functional magnetic resonance imaging'/exp OR 'functional magnetic resonance imaging':ab,ti OR 'nuclear magnetic resonance imaging'/exp OR 'nuclear magnetic resonance imaging':ab,ti OR 'echography'/exp OR echography:ab,ti OR 'dual energy x ray absorptiometry'/exp OR 'dual energy x ray absorptiometry':ab,ti OR 'muscle'/exp OR 'muscle mass'/exp OR 'lean mass'/exp OR 'fat free mass'/exp OR 'fat mass'/exp OR 'sarcopenia'/exp OR 'muscle*':ab,ti OR 'lean mass':ab,ti OR 'fat free mass':ab,ti OR 'fat mass':ab,ti OR 'sarcopenia':ab,ti OR 'obesity'/exp OR obes*:ti,ab OR 'FSS':ab,ti OR 'fatigue*':ab,ti OR 'Shortened fatigue questionnaire':ab,ti OR 'SFQ':ab,ti OR 'FAS':ab,ti OR 'multidimensional assessment of fatigue scale':ab,ti OR 'MAF':ab,ti OR 'tiredness':ab,ti OR 'fatigue'/exp OR 'chronic fatigue syndrome'/exp OR 'chronic fatigue syndrome':ab,ti OR 'International Classification of Functioning, Disability and Health'/exp OR 'International Classification of Functioning, Disability and Health':ab,ti OR 'cognition'/exp OR 'cognition':ab,ti OR 'sensory'/exp OR 'sensory':ab,ti OR 'voice'/exp OR 'voice':ab,ti OR 'respiratory function'/exp OR 'respiratory function':ab,ti OR 'immunity'/exp OR 'immunity':ab,ti OR 'cardiovascular system'/exp OR 'cardiovascular system':ab,ti OR 'endocrine system'/exp OR 'endocrine system':ab,ti OR 'digestive system'/exp OR 'digestive system':ab,ti OR 'genital system'/exp OR 'genital system':ab,ti OR 'movement (physiology)'/exp OR 'movement':ab,ti OR 'skin'/exp OR 'skin':ab,ti OR 'nervous system'/exp OR 'nervous system':ab,ti OR 'eye'/exp OR 'eye':ab,ti OR 'learning'/exp OR 'learning':ab,ti OR 'knowledge'/exp OR 'knowledge':ab,ti OR 'interpersonal communication'/exp OR 'interpersonal communication':ab,ti OR 'mobility'/exp OR 'mobility':ab,ti OR 'joint mobility'/exp OR 'joint mobility':ab,ti OR 'self care'/exp OR 'self care':ab,ti OR

'household'/exp OR 'household':ab,ti OR 'empathy'/exp OR 'empathy':ab,ti OR 'education'/exp OR 'education':ab,ti OR 'human relation'/exp OR 'human relation':ab,ti OR 'work'/exp OR 'work':ab,ti OR 'employment'/exp OR 'employment':ab,ti OR 'daily life activity'/exp OR 'daily life activity':ab,ti OR 'social participation'/exp OR 'social participation':ab,ti OR 'participation'/exp OR 'participation':ab,ti OR 'social support'/exp OR 'social support':ab,ti OR 'family'/exp OR 'family':ab,ti OR 'attitude'/exp OR 'attitude':ab,ti OR 'physical disease by body function'/exp OR 'physical disease by body function':ab,ti OR 'body system':ab,ti OR 'international classification of functioning disability and health'/exp OR 'psychosocial functioning'/exp OR 'psychosocial funct*' OR 'social interaction'/exp OR 'social interaction' OR 'habits':ab,ti OR 'coping':ab,ti OR 'lifestyle':ab,ti OR 'social status':ab,ti OR 'coping behavior'/exp OR 'habit'/exp OR 'lifestyle'/exp)

Search string Scopus

(TITLE-ABS-KEY ("Liped*" OR "Lipoed*" OR "Lipolymphedema")) AND (TITLE-ABS-KEY ("nutri*" OR "malnutrition" OR "Nutritional Deficienc*" OR "Undernutrition" OR "Malnourish*" OR "Avitaminosis" OR "Deficiency Diseas*" OR "Protein Deficienc*" OR "Protein-Energy Malnutrition" OR "Overnutrition" OR "Hypervitaminosis A" OR "Feeding and Eating Dis*" OR "Binge-Eating Dis*" OR "Undernourish*" OR "Malnourish*" OR "physical funct*" OR "physical Fitness" OR "Cardiorespiratory Fitness" OR "physical ability*" OR "physical health" OR "functional ability*" OR "Exercise*" OR "Physical Activit*" OR "rand 36" OR "SF 36" OR "36 Item Short Form Health Survey" OR "Short Form*" OR "EQ-5D*" OR "EuroQuol 5D" OR "Musculoskeletal Function Assessment" OR "MFA" OR "SMFA" OR "WHOQOL*" OR "Lower Extremity Functional Scale" OR "LEFS" OR "Short Questionnaire to Assess Health-enhancing physical activity" OR "SQUASH" OR "LASA Physical Activity Questionnaire" OR "LAPAQ" OR "Pedometer" OR "Patient Specific Complaints List" OR "PSK" OR "Multidimensional Fatigue Index" OR "MFI" OR "MVI" OR "MVI 20" OR "MFI 20" OR "Central Sensitization Inventory" OR "CSI" OR "6MWT" OR "walk* test" OR "6MWD" OR "walk* distance" OR "steep ramp test" OR "srt" OR "Quadriceps*" OR "Quadriceps Femoris" OR "Timed Chair Stand Test" OR "Sit to Stand" OR "TCST" OR "FTSTS" OR "FRSTS" OR "10MWT" OR "Exercise Test" OR "Hand Strength" OR "handgrip" OR "*grip strength" OR "hand held dynamometer" OR "graps strength" OR "depress*" OR "stress" OR "psychological funct*" OR "kinesiophobia" OR "fear*" OR "Psychosocial Funct*" OR "panic" OR "mental health" OR "Psych*" OR "mental" OR "*Pain" OR "pain*" OR "Physical Suffering" OR "Ache*" OR "Visual Analogue Scale" OR "VAS" OR "numeric pain rating scale" OR "numeric rating scale" OR "NPRS" OR "NRS" OR "Muscle fat*" OR "muscle-to-fat*" OR "fat-muscle*" OR "fat-to-muscle*" OR "fat ratio*" OR "muscle*" OR "body composition" OR "muscle tissue*" OR "tissue distribution" OR "adipose tissue*" OR "Magnetic Resonance Imaging" OR "Ultrasonography" OR "Absorptiometry " OR "ultrasound" OR "dual energy X-ray absorptiometry" OR "echography" OR "BIA" OR "bio electrical impendance analysis" OR "bio impendance analysis" OR "Lean mass" OR "Fat-free*" OR "Fat mass" OR "sarcopenia" OR "adiposity" OR "obes*" OR "FSS" OR "fatigue*" OR "Shortened fatigue questionnaire" OR "SFQ" OR "FAS" OR "multidimensional assessment of fatigue scale" OR "MAF" OR "tiredness" OR "cognition" OR "sensation" OR "international classification of functioning" OR "voice" OR "respiratory" OR "immune system" OR "cardiovascular system" OR "endocrine system" OR "digestive system" OR "genitalia" OR "movement" OR "skin" OR "nervous system" OR "eye" OR "learning" OR "knowledge" OR "communication" OR "education" OR "mobility" OR "self care" OR "household" OR "environment" OR "interpersonal relation" OR "activities" OR "participation" OR "body function" OR "body system" OR "work" OR "attitude" OR "employment" OR "family" OR "International Classification of Functioning Disability and Health" OR "Psychosocial Functioning" OR "Social Interaction" OR "International Classification of Functioning Disability and Health" OR "Psychosocial Functioning" OR "Social Interaction" OR "habits" OR "coping" OR "lifestyle" OR "social status"))

Search string BASE & science.gov & clinicaltrail.gov & WHO ICTRP
"Lipedema" OR "Lipoedema" OR "Lipolymphedema"
Search string Lipedema Foundation LEGATO Lipedema Library
("Lipedema" OR "Lipoedema" OR "Lipolymphedema") AND ("nutri*" OR "physical funct*" OR "physical Fitness" OR "Exercise*" OR "psychological funct*" OR ICF OR "Psychosocial Funct*" OR "*Pain" OR "pain*" OR "International Classification of Functioning Disability and Health")
