# Supplementary material for: Functioning of People with Lipoedema According to All Domains of the International Classification of Functioning, Disability and Health: A Scoping Review
Source: Int J Environ Res Public Health. 2023 Jan 21;20(3):1989. doi: 10.3390/ijerph20031989 (PMC9915552; doi:10.3390/ijerph20031989)
Supplement: Supplementary file 1 [file ijerph-20-01989-s001.zip › Supplementary files B L.M. Kloosterman.pdf]

**Supplementary Table S2. Study characteristics**

| Author(s), year, design, country | Population and lipoedema characteristics | Outcomes | | |
|---|---|---|---|---|
| | | **ICF** | **Lipoedema group(s)** | **Control group(s)** |
| Amann-Vesti et al., 2001<br><br>Cross-sectional study<br><br>Switzerland | N = 24<br><br>Lipoedema group n = 12<br>Age in years: mean (range) 43.7 (21-76)<br><br>Lipoedema location: % of population<br>Calves: 33.3%<br>Calves and thighs: 58.3%<br>Thighs: 8.33%<br><br>Control group (healthy) n = 12<br>Age in years: mean (range) 34.6 (24-56) | S420<br><br><br><br><br><br>S420<br><br><br><br><br><br>S420 | Capillary diameter (μm): mean ± sd<br>*Method: Fluorenscence microlymphography*<br>Thigh: 53.3 ± 19.5<br>Medial ankle: 48.7 ± 22.1<br>Dorsum of the foot: 51.1 ± 4.6<br><br>Network extension (μm): mean ± sd<br>*Method: Fluorenscence microlymphography*<br>Thigh: 6.7 ± 3.5<br>Medial ankle: 5.8 ± 4.7<br>Dorsum of the foot: 5.7 ± 2.0<br><br>Number of microaneurysms at thigh (n=8): mean (range)<br>*Method: Fluorenscence microlymphography*<br>7.6 (2-14) | Capillary diameter (μm): mean ± sd<br>*Method: Fluorenscence microlymphography*<br>Thigh: 50.0 ± 5.6<br>Medial ankle: 49.0 ± 5,1<br>Dorsum of the foot: 46.6 ± 21.1<br><br>Network extension (μm): mean ± sd<br>*Method: Fluorenscence microlymphography*<br>Thigh: 3.8 ± 0.8<br>Medial ankle: 5.6 ± 1.1<br>Dorsum of the foot: 5.2 ± 1.3<br><br>Number of microaneurysms at thigh: mean (range)<br>*Method: Fluorenscence microlymphography*<br>0.08 (0-1) |
| Amato et al., 2020<br><br>Cross-sectional study<br><br>Brazil | N = 109<br><br>Lipoedema group n = 59<br>Age in years: mean (range) 42 (19-77)<br>Control group n = 50<br>Age in years: mean (range) 45.7 (24-79) | B530 | BMI (kg/cm$^2$): mean (range)<br>29.68 (20.4-46.2) | BMI (kg/cm$^2$): mean (range)<br>29.1 (20.4-45.6) |

| | | | | |
|---|---|---|---|---|
| Amato et al., 2021<br><br>Cross-sectional study<br><br>Brazil | N = 89<br><br>Lipoedema group n = 62<br>Age in years: mean ± sd: 46.177 ± 3.587<br><br>Lipoedema type: % of population<br>I: 16.1%<br>II: 27.4%<br>III: 56.4%<br><br>Lipoedema stage: % of population<br>I: 40.3%<br>II: 24.2%<br>III: 25.8%<br>IV: 9.6%<br><br>Control group (with varicose veins) n = 27<br>Age in years: mean ± sd 47.963 ± 4.467 | B530<br><br>S810<br><br>P | BMI (kg/cm²): mean ± sd<br>30.0 ± 1.4<br><br>Thickness cutis and subcutaneous tissue (mm): mean ± sd<br>*Method: Ultrasound*<br>thigh right 20.9 ± 0.167<br>thigh left 20.32 ± 0.149<br>lateral leg right 12.85 ± 0.137<br>lateral leg left 13.77 ± 0.331<br>pretibial region right 16.17 ± 0.2<br>pretibial region left 16.5 ± 0.205<br>Supramalleolar right 12.08 ± 0.184<br>supramalleolar left 11.8 ± 0.172<br><br>Varicose veins identified: % of population<br>45.1% | BMI (kg/cm²): mean ± sd<br>27.4 ± 1.5<br><br>Thickness cutis and subcutaneous tissue (mm): mean ± sd<br>*Method: Ultrasound*<br>thigh right 12.67 ±0.18<br>thigh left 12.38 ± 0.151<br>lateral leg right 6.8 ± 0.083<br>lateral leg left 6.4 ± 0.078<br>pretibial region right 8.3 ± 0.092<br>pretibial region left 8.45 ± 0.097<br>supramalleolar right 6.09 ± 0.109<br>supramalleolar left 6.6 ± 0.12<br><br>Varicose veins identified: % of population<br>100.0% |
| Angst et al., 2021<br><br>Cross-sectional study<br><br>Switzerland | N = 189<br><br>Lipoedema group n = 112<br>Age in years: mean ± sd 45.4 ± 13.1<br><br>Control group (fibromyalgia) n= 77<br>Age in years: mean ± sd 49.3 ± 9.5 | B4550<br><br>B530<br><br>D850<br><br>D920<br><br>P | 6MWT (meters) (n = 38): mean ± sd<br>437 ± 128<br><br>BMI (kg/m²): mean ± sd<br>33.7 ± 7.5<br><br>Working place (hours/week): % of population<br>0: 12.9%<br>1-21: 17.8%<br>22-41: 37.6%<br>≥ 42: 31.7%<br><br>Sports activities (hours/week): % of population<br>0: 19.8%<br>1-2: 43.3%<br>>2: 36.8%<br><br>Education: % of population<br>Basic school 11.8%<br>Vocational training 56.4%<br>College/high/technical school 17.3%<br>University 14.5%<br>Living situation: % of population | 6MWT (meters) (n = 38): mean ± sd<br>425 ± 133<br><br>BMI (kg/m²): mean ± sd<br>27.4 ± 5.7<br><br>Working place (hours/week): % of population<br>0: 44.1%<br>1-21: 14.7%<br>22-41: 25.0%<br>≥ 42: 16.2%<br><br>Sports activities (hours/week): % of population<br>0: 48.1%<br>1-2: 37.7%<br>>2: 14.3%<br><br>Education: % of population<br>Basic school 18.2%<br>Vocational training 57.1%<br>College/high/technical school 11.7%<br>University 13.0%<br>Living situation: % of population |

| | | | | |
|---|---|---|---|---|
| | | P | Alone 28.4%<br>With partner 61.5%<br>With other persons 10.1% | Alone 20.8%<br>With partner 63.6%<br>With other persons 15.6% |
| | | P | SF -36 (0-100 each domain): (mean ± sd)<br>Physical functioning 68.1 ± 22.5<br>Role physical 61.7 ± 26.4<br>Bodily pain 43.4 ± 23.8<br>General health 53.8 ± 18.6<br>Vitality 43.3 ± 19.6<br>Social functioning 65.5 ± 26.9<br>Role emotional 68.6 ± 27.8<br>Mental health 61.1 ± 19.0<br>PCS 41.9 ± 9.1<br>MCS 42.2 ± 12.2 | SF -36 (0-100 each domain): (mean ± sd)<br>Physical functioning 44.9 ± 19.7<br>Role physical 29.7 ± 19.1<br>Bodily pain 19.4 ± 13.7<br>General health 35.4 ± 14.7<br>Vitality 25.8 ± 16.9<br>Social functioning 38.0 ± 24.7<br>Role emotional 40.2 ± 29.2<br>Mental health 40.1 ± 19.6<br>PCS 32.9 ± 6.5<br>MCS 30.3 ± 11.6 |
| | | P | Smoking habits: % of population<br>11.3% | Smoking habits: % of population<br>29.9% |
| | | P | Comorbidities (number): % of population<br>None 9.1%<br>1: 17.3%<br>2: 21.8%<br>3: 24.5%<br>4: 11.8%<br>≥5: 15.5% | Comorbidities (number): % of population<br>None 3.9%<br>1: 7.8%<br>2: 10.48%<br>3: 23.4%<br>4: 22.1%<br>≥5: 32.4% |
| Atan et al., 2020<br><br>Randomised controlled trial<br><br>Turkey | N = 31<br><br>Lipoedema group 1 n = 11<br>Age in years: mean ± sd: 58.36 ± 8.98<br>Lipoedema group 2 n = 10<br>Age in years: mean ± sd: 58.90 ± 7.70<br>Lipoedema group 3 n = 10<br>Age in years: mean ± sd: 60.10 ± 5.50<br><br>Lipoedema stage: % of population<br>III: 29.0%<br>IV: 71.0%<br><br>Duration of disease in years: mean ± sd<br>Lipoedema group 1: 11.36 ± 2.83 | B152 | Beck Depression Inventory (0-63): mean ± sd<br>Group 1: 24.18 ± 9.80<br>Group 2: 25.20 ± 6.47<br>Group 3: 23.60 ± 7.18 | n/a |
| | | B280 | Pain VAS: mean ± sd<br>Group 1: 7.73 ± 1.67<br>Group 2: 8.30 ± 1.70<br>Group 3: 7.90 ± 1.47 | |
| | | B4550 | 6MWT (meters): mean ± sd<br>Group 1: 304.81 ± 105.39<br>Group 2: 321.70 ± 92.85<br>Group 3: 328.30 ± 59.80 | |

| | | |
|---|---|---|
| Lipoedema group 2: 11.60 ± 3.13<br>Lipoedema group 3: 10.90 ± 2.76 | B4552 | Fatigue severity scale: mean ± sd<br>Group 1: 5.67 ± 1.26<br>Group 2: 5.77 ± 0.67<br>Group 3: 4.80 ± 1.10 |
| | B530 | BMI (kg/m$^2$): mean ± sd<br>Group 1: 43.49 ± 4.44<br>Group 2: 40.66 ± 3.81<br>Group 3: 42.06 ± 5.76 |
| | B530 | Waist-to-height ratio : mean ± sd<br>Group 1: 0.73 ± 0.05<br>Group 2: 0.66 ± 0.05<br>Group 3: 0.71 ± 0.07 |
| | B530 | Waist-to-hip ratio: mean ± sd<br>Group 1: 0.86 ± 0.06<br>Group 2: 0.82 ± 0.04<br>Group 3: 0.84 ± 0.06 |
| | D230 | IPAQ total score (MET/min): mean ± sd<br>Group 1: 381.63 ± 455.16<br>Group 2: 233.85 ± 149.44<br>Group 3: 425.70 ± 286.92 |
| | P | Education level: % of population<br>Low: 3.0%<br>Intermediate: 94.0%<br>High: 3.0% |
| | P | SF -36 (0-100 each domain): mean ± sd<br>Physical functioning<br>Group 1: 31.36 ± 12.26<br>Group 2: 29.00 ± 15.23<br>Group 3: 32.50 ± 27.30<br>Role limitations due to physical health<br>Group 1: 11.36 ± 25.89<br>Group 2: 12.50 ± 17.67<br>Group 3: 21.00 ± 34.05<br>Role limitations due to emotional problems<br>Group 1: 15.13 ± 17.39 |

| | | | | |
|---|---|---|---|---|
| | | | Group 2: 13.33 ± 23.30<br>Group 3: 13.33 ± 32.20<br>Energy/fatigue:<br>Group 1: 31.36 ± 16.13<br>Group 2: 36.50 ± 24.04<br>Group 3: 38.20 ± 13.54<br>Social functioning<br>Group 1: 37.50 ± 24.36<br>Group 2: 35.00 ± 17.48<br>Group 3: 47.60 ± 18.61<br>Pain<br>Group 1: 27.25 ± 16.26<br>Group 2: 25.25 ± 18.33<br>Group 3: 30.25 ± 14.59<br>General health<br>Group 1: 21.81 ± 15.85<br>Group 2: 30.00 ± 15.81<br>Group 3: 32.50 ± 16.37<br>Emotional well-being:<br>Group 1: 44.00 ± 18.24<br>Group 2: 43.60 ± 10.05<br>Group 3: 42.90 ± 20.34<br>Health change<br>Group 1: 35.00 ± 24.15<br>Group 2: 36.36 ± 17.18<br>Group 3: 38.50 ± 22.11 | |
| Beltran et al., 2016 | N = 272 | B134 | Difficulty sleeping: % of population 21.3% | Difficulty sleeping: % of population 23.4% |
| Cross-sectional study | Lipoedema group n = 160<br>Age in years: mean ± sd 50 ± 14 | B140 | Poor concentration: % of population 19.4% | Poor concentration: % of population 29.8% |
| | F/M: % of population 99.4%/0.6% | B265 | Numbness: % of population 21.3% | Numbness: % of population 23.4% |
| USA | Lipoedema stage: % of population<br>I: 16.0% | B280 | Muscles aches: % of population 26.3% | Muscles aches: % of population 26.6% |
| | II: 49.0%<br>III: 30.0% | B28010 | Headaches: % of population 21.3% | Headaches: % of population 23.4% |
| | IV: 4.0% | B28013 | Low back pain: % of population 20.0% | Joint aches: % of population 27.7% |
| | Control group (Decrums Disease) n = 94<br>Age in years: mean ± sd 49 ± 12 | B28016 | Joint aches: % of population 30.0% | Low back pain: % of population 24.4% |
| | F/M: % of population 84.0%/16.0% | B530 | BMI (kg/m$^2$): mean ± sd 39.0 ± 12.0 | BMI (kg/m$^2$): mean ± sd 33.0 ± 8.0 |

| | | | | |
|---|---|---|---|---|
| | | B420 | Blood pressure (mmHg): mean ± sd<br>Systolic: 133 ± 17<br>Diastolic: 78 ± 12 | Blood pressure (mmHg): mean ± sd<br>Systolic: 136 ± 19<br>Diastolic: 79 ± 10 |
| | | B435 | Swelling: % of population 39.4% | Swelling: % of population 29.8% |
| | | B4552 | Fatigue: % of population 37.0% | Fatigue: % of population 38.1% |
| | | B460 | Shortness of breath: % of population 12.5% | Shortness of breath: % of population 27.7% |
| | | B525<br>B525 | Constipation: % of population 20.6%<br>Diarrhea: % of population 14.4% | Constipation: % of population 22.3%<br>Diarrhea: % of population 23.4% |
| | | B620 | Nocturia: % of population 21.6% | Nocturia: % of population 15.0% |
| | | B710 | Beighton score (0-9) (≥ 5): % of population ± sd 58.0 ± 0.5% | Beighton score (0-9) (≥ 5): % of population ± sd 23.0 ± 0.4% |
| | | B730 | Muscle weakness: % of population 18.1% | Muscle weakness: % of population 23.4% |
| | | B820 | Easy bruising: % of population 28.1% | Easy bruising: % of population 15.0% |
| | | B840 | Itching: % of population 21.3% | Itching: % of population 23.4% |
| | | P | Comorbidities: % of population ± sd<br>Arthritis 15.0 ± 0.4<br>Diabetis 6.0 ± 0.2<br>Fibromyalgia 10.0 ± 0.3<br>Hypertension 18.0 ± 0.4<br>Hypothyroidism 27.0 ± 0.4<br>Lipomas 0<br>Migraines 7.0 ± 0.3<br>Depression 17.5<br>Anxiety 18.1<br>Visible veins on legs 25.0 | Comorbidities: % of population ± sd<br>Arthritis 15.0 ± 0.4<br>Diabetis 16.0 ± 0.4<br>Fibromyalgia 28.0 ± 0.4<br>Hypertension 21.0 ± 0.4<br>Hypothyroidism 26.0 ± 0.4<br>Lipomas 20.0 ± 0.4<br>Migraines 21.0 ± 0.4<br>Depression 24.5<br>Anxiety 21.2<br>Visible veins on legs 13.8 |
| | | P | Race: % of population<br>Caucasian: 97.0%<br>Black/African American: 3.0% | Race: % of population<br>Caucasian: 100.0%<br>Black/African American: 0.0% |
| Buso et al., 2022 | N = 45 | B280 | Pain: % of population 100.0% | n/a |

| | | | |
|---|---|---|---|
| Cross-sectional study<br><br>Switzerland | Age in years: mean ± sd<br>39.0 ± 10.5<br><br>Lipoedema stage: % of population<br>I: 22.0%<br>II: 49.0%<br>III: 29.0%<br><br>Lipoedema type: % of population<br>I: 4.0%<br>II: 16.0%<br>III: 49.0%<br>IV: 29.0%<br><br>Duration of disease in years: mean ± sd<br>23.4 ± 11.8<br><br>Age at onset in years: mean ± sd<br>15.9 ± 8.4 | B435 | Lymphatic functioning left and right leg: median (IQR)<br>*Method: Indocyanine green lymphography*<br>Distance covered (cm) by dye after 10 min.: 0.46 (0.35-0.75)<br>Distance covered (cm) by dye after 25 min.: 0.79 (0.54-0.85)<br>Distance covered (cm) after MLD: 0.87 (0.84-0.89)<br>Dye did not reach groin within 25 min.: % of legs 55.6% |
| | | S420 | Lymphatic anatomy: % of legs<br>*Method: Indocyanine green lymphography*<br>Linear patter: 100.0%<br>Dermal rerouting: 2.2% |
| | | B4552 | Fatigue: % of population 15.0% |
| | | B530 | BMI ($kg/m^2$): median (IQR) 27.57 (25.64-32.71) |
| | | B530 | Body composition<br>*Method: Dual-energy X-ray absorptiometry*<br>Fat mass index (FMI) ($kg/m^2$): median (IQR) 11.92 (1.00-15.15)<br>Lean mass index (LMI) ($kg/m^2$): median (IQR) 15.72 (14.09-16.74)<br>Appendicular LMI ($kg/m^2$): mean ± sd 7.40 ± 1.05<br>Total leg fat per BMI ($kg/(kg/m^2)$): mean ± sd 0.55 ± 0.11<br>Total leg fat per FMI ($kg/(kg/m^2)$): mean ± sd 1.27 ± 0.18<br>Total leg lean per BMI ($kg/(kg/m^2)$): mean ± sd 0.55 ± 0.07<br>Total leg lean per LMI ($kg/(kg/m^2)$): median (IQR) 1.02 (0.95-1.11)<br>Android/gynoid fat ratio: mean ± sd 0.86 ± 0.13<br>Visceral adipose tissue per FMI: median (IQR) 29.15 (16.63-54.46) |
| | | B780 | Heaviness: % of population 69.0% |
| | | B820 | Easy bruising: % of population 91.0% |
| | | E580 | Compressive garments: % of population 58.0% |
| | | E580 | Duration of compressive therapy in months: median (IQR) 4.0 (2.0-8.0) |

| | | | | |
|---|---|---|---|---|
| Cannataro et al., 2021<br><br>Case report<br><br>Italy | N = 1<br><br>Age in years: 32<br><br>Lipoedema stage: I<br>Lipoedema type: IV and V | B134<br><br>B280<br><br><br>B4301<br>B545<br>B545<br>B545<br>B598<br>B598<br>B598<br>B598<br>B610<br>B610<br><br>P | SQS (0-84): 37<br><br>Pain VAS (0-10): 9.2<br><br>Biochemical parameters:<br>Hb (g/dL): 13.9 (normal 12.0-15.5)<br>Serum iron (μg/dL): 102 (normal 6-170)<br>Serum calcium (mmol/L): 2.4 (normal 2.2-2.7)<br>Serum potassium (mmol/L): 3.74 (normal 3.6-5.2)<br>Glycemia (mg/dL): 99 (normal 70-100)<br>Insulin (mI/L): 29.3 (normal 4-25)<br>HOMA-IR: 7.16 (normal <2.60)<br>HbA1C (mmol/mol): 32 (normal 20-38)<br>Creatinine (mg/dL): 0.93 (normal 0.6-1.2)<br>Uric acid (mg/dL): 2.5 (normal 2.7-7.3)<br><br>WOMAC: 45 | n/a |
| Cellina et al., 2020<br><br>Cross-sectional study<br><br>Italy | N = 22<br><br>Lipoedema group n = 11<br>Age in years: mean ± sd<br>33.2 ± 7<br><br>Duration of disease in years: mean ± sd<br>13.3 ± 7.4<br><br>Control group (Lipolymphoedema) n = 11<br>Age in years: mean ± sd<br>39.0 ± 12.0 | B435<br><br><br><br><br>S420<br><br><br><br><br>B530<br><br><br>B530<br><br>P | Lymphatic functioning: % of population<br>*Method: Non-contrast Magnetic Resonance Lymphography*<br>Fluid infiltration of the subcutaneous fat: 18.2%<br>Epifascial fluid collection: 0.0%<br><br>Lymphatic anatomy: % of population<br>*Method: Non-contrast Magnetic Resonance Lymphography*<br>Normal appearance of the main lymphatic trunks: 100.0%<br>Dilated distal lymphatic vessels: 18.2%<br><br>BMI (kg/m$^2$): mean ± sd<br>31.2 ± 1.1<br><br>Enlarged subcutaneous fat: % of population 100.0%<br><br>Signs of vascular stasis: % of population 36.3% | Lymph function: % of population<br>*Method: Non-contrast Magnetic Resonance Lymphography*<br>Fluid infiltration of the subcutaneous fat:100.0%<br>Epifascial fluid collection: 10.0%<br><br>Lymphatic anatomy: % of population<br>*Method: Non-contrast Magnetic Resonance Lymphography*<br>Normal appearance of the main lymphatic trunks: 100.0%<br>Dilated distal lymphatic vessels: 90.9%<br><br>BMI (kg/m$^2$): mean ± sd<br>31.4 ± 1.2<br><br>Enlarged subcutaneous fat: % of population 100.0%<br><br>Signs of vascular stasis: % of population 45.4% |

| | | | | |
|---|---|---|---|---|
| Crescenzi et al., 2019<br><br>Cross-sectional study<br><br>USA | N = 136<br><br>Lipoedema group: n = 110<br><br>Control group 1 (Decrums Disease) n = 25<br>Age in years: mean ± sd 56.9 ± 8.6<br><br>Control group 2 (healthy) n = 21<br>Age in years: mean ± sd 45.7 ± 11.1<br><br>Lipoedema stage: % of population<br>I: 9.0%<br>II: 44.0%<br>III: 47.0% | B530 | BMI (kg/cm$^2$): mean ± sd<br>37.5 ± 10.0 | BMI (kg/cm$^2$): mean ± sd<br>1: 37.6 ± 8.2<br>2: 28.4 ± 4.1 |
| | | B545 | Extracellular water levels ($R_0$ (Ω)/ $R_{00}$(Ω): mean ± sd<br>*Method: Bioimpedance spectroscopy*<br>Arms: $R_0$(Ω) 334.4 ± 39.0/ $R_{00}$(Ω) 239.6 ± 40.1<br>Legs: $R_0$(Ω) 241.7 ± 53.8/ $R_{00}$(Ω) 185.2 ± 40.2 | Extracellular water levels ($R_0$ (Ω)/ $R_{00}$(Ω): mean ± sd<br>*Method: Bioimpedance spectroscopy*<br>1: Arms: $R_0$(Ω) 328.4± 43.2/ $R_{00}$(Ω) 238.2 ± 38.8<br>Legs: $R_0$(Ω) 263.0 ± 56.6/ $R_{00}$(Ω) 199.2 ± 45.1<br>2: Arms: $R_0$(Ω) 341.8 ± 32.6/ $R_{00}$(Ω) 244.0 ± 28.7<br>Legs: $R_0$(Ω) 294.8 ± 32.8 / $R_{00}$(Ω) 218.1 ± 25.4 |
| | | P | Race: % of population<br>White: 95.0%<br>Black: 5.0% | Race: % of population<br>1: White: 92.0%<br>American Indian or Alaskan Native: 4.0%<br>Declined to comment: 4.0%<br>2: White: 100.0% |
| Dadras et al., 2017<br><br>Retrospective cohort<br><br>Germany | N = 25<br><br>Age in years: median (range) 45 (23-64)<br><br>Lipoedema stage: % of population<br>II: 44.0%<br>III: 52.0% | B1801 | Cosmetic impairment VAS (0-10): mean ± sd 8.98 ± 0.81 | n/a |
| | | B2702 | Sensitivity to pressure VAS (0-10): mean ± sd 7.38 ± 1.77 | |
| | | B280 | Spontaneous pain VAS (0-10): mean ± sd 7.2 ± 1.46 | |
| | | B530 | BMI (kg/m$^2$): mean (range)<br>35.3 (24.5-50.6) | |
| | | B780 | Feeling of tension VAS (0-10): mean ± sd 7.52 ± 1.36 | |
| | | B820 | Bruising VAS (0-10): mean ± sd 6.96 ± 1.58 | |
| | | P | Impairment of quality of life VAS (0-10): mean ± sd 8.38 ± 1.06 | |
| | | E580 | CDT* score: mean ± sd 20.48±4.13<br>**number of manual lymphatic drainage sessions per month and the number of hours spent wearing compression garments per day.* | |

| | | | | |
|---|---|---|---|---|
| Dietzel et al., 2015<br><br>Cross-sectional study<br><br>Germany | N = 127<br><br>Lipoedema group n = 49<br>BMI 18.5-24.9: n= 8<br>Age in years: mean ± sd 37.38 ± 10.99<br>BMI 25-29.5: n = 18<br>Age in years: mean ± sd 48.89 ± 8.86<br>BMI 30-39.9: n = 13<br>Age in years: mean ± sd 46.54 ± 9.74<br>BMI ≥ 40: n = 10<br>Age in years: mean ± sd 49.30 ± 6.55<br><br>Control group (healthy) n = 78<br>BMI 18.5-24.9: n = 16<br>Age in years: mean ± sd 37.75 ± 8.84<br>BMI 25-29.5: n = 36<br>Age in years: mean ± sd 43.94 ± 10.32<br>BMI 30-39.9: n = 26<br>Age in years: mean ± sd 45.23 ± 9.44<br>BMI ≥ 40: n = 0 | B530 | BMI (kg/$m^2$): mean ± sd<br>18.5-24.9: 22.93 ± 1.44<br>25-29.5: 27.99 ± 1.46<br>30-39.9: 34.59 ± 3.62<br>≥ 40: 44.33 ± 3.95 | BMI (kg/$m^2$): mean ± sd<br>18.5-24.9: 23.01 ± 1.36<br>25-29.5: 27.53 ± 1.22<br>30-39.9: 32.95 ± 2.05<br>≥ 40: 0 |
| | | B530 | Leg-trunk index by BMI: mean ± sd<br>18.5-24.9: 0.058 ± 0.018<br>25-29.5: 0.036 ± 0.012<br>30-39.9: 0.027 ± 0.007<br>≥ 40: 0.024 ± 0.005 | Leg-trunk index by BMI: mean ± sd<br>18.5-24.9: 0.038 ± 0.008<br>25-29.5: 0.027 ± 0.007<br>30-39.9: 0.022 ± 0.004<br>≥ 40: 0 |
| | | B530 | Android-gynoid index by BMI: mean ± sd<br>18.5-24.9: 0.011 ± 0.003<br>25-29.5: 0.014 ± 0.002<br>30-39.9: 0.013 ± 0.002<br>≥ 40: 0.010 ± 0.002 | Android-gynoid index by BMI: mean ± sd<br>18.5-24.9: 0.015 ± 0.003<br>25-29.5: 0.017 ± 0.004<br>30-39.9: 0.015 ± 0.003<br>≥ 40: 0 |
| Di Renzo et al., 2021<br><br>Controlled clinical trial<br><br>Italy | N = 29<br><br>Lipoedema group 1 n = 14<br>Lipoedema group 2 n = 15<br><br>Age in years: all < 65 year.<br><br>Lipoedema stage: % of population<br>I: 14.3%<br>II: 42.8%<br>III: 28.6%<br>IV: 14.3% | B280 | Pain VAS (0-100): mean ± sd<br>64.7 ± 18.3 | n/a |
| | | B530 | BMI (kg/$m^2$): mean ± sd<br>Group 1: 35.50 ± 12.17<br>Group 2: 27.52 ± 5.22 | |
| | | B530 | Waist-to-hip ratio: mean ± sd<br>Group 1: 0.72 ± 0.07<br>Group 2: 0.78 ± 0.08 | |
| | | B530 | Body composition: mean ± sd LG 1/ LG 2<br>*Method: Dual-energy X-ray absorptiometry*<br>Fat mass, trunk (kg): 14.09 ± 5.97 / 18.66 ± 11.71<br>Fat mass, android (kg): 2.26 ± 1.14 / 3.08 ± 2.06<br>Fat mass, gynoid (kg): 5.43 ± 1.74 / 7.81 ± 4.33<br>Fat mass, total body (kg): 29.2 ± 9.8 / 42.02 ± 21.94<br>Lean mass, arms (kg): 4.49 ± 0.97 / 4.41 ± 0.78<br>Lean mass, legs (kg): 14.82 ± 2.58 / 17.13 ± 2.83<br>Lean mass, trunk (kg): 19.31 ± 2.88 / 21.42 ± 4.22<br>Lean mass, total body (kg): 41.74 ± 6.27 / 46.65 ± 7.99 | |

Total mass, arms (kg): 8.01 ± 1.82 / 8.74 ± 2.54
Total mass, legs (kg): 26.65 ± 5.58 / 36.15 ± 10.62
Total mass, trunk (kg): 34.13 ± 7.73 / 40.86 ± 15.33
Total mass, android (kg): 5.12 ± 1.45 / 6.38 ± 2.63
Total mass, gynoid (kg): 12.12 ± 2.4 / 15.27 ± 5.58
Fat region (%), arms: 39.75 ± 5.61 / 44.16 ± 10.39
Fat region (%), legs: 40.6 ± 6.32 / 48.19 ± 9.48
Fat region (%), trunk: 39.92 ± 9.40 / 41.61 ± 13.91
Fat region (%), android: 42.08 ± 11.31 / 43.04 ± 16.63
Fat region (%), gynoid: 43.95 ± 6.90 / 48.20 ± 11.03
Fat region (%), total body: 39.02 ± 7.13 / 42.78 ± 11.63
Intermuscular adipose tissue: 1.14 ± 0.43 / 1.37 ± 0.54

B540 Oxygen consumption (ml/min): mean ± sd
*Method: Indirect calorimetry*
Group 1: 217.14 ± 46.08
Group 2: 218.87 ± 31.61

B540 Cardon dioxide production (ml/min): mean ± sd
*Method: Indirect calorimetry*
Group 1: 173.57 ± 41.52
Group 2: 182.13 ± 29.55

B545 Resting energy expenditure (kcal): mean ± sd
*Method: Indirect calorimetry*
Group 1: 1479.21 ± 321.51
Group 2: 1508.00 ± 247.11

B545 Total body water (L): mean ± sd
*Method: Bioelectrical impedance analysis*
Group 1: 37.99 ± 5.84
Group 2: 34.89 ± 4.66

B545 Extracellular water (L): mean ± sd
*Method: Bioelectrical impedance analysis*
Group 1: 18.88 ± 3.37
Group 2: 15.80 ± 2.08

P EQ-5D: mean ± sd
Total score: 8.3 ± 1.8

| | | | | |
|---|---|---|---|---|
| Erbacher et al., 2020<br><br>Cross-sectional study<br><br>Germany | N = 150<br><br>Age in years: mean (range)<br>43.58 (17.0-69.0) | B280 | Pain VAS max. / min.: % of population<br>0: 0.0% / 28.7%<br>1: 0.0% / 6.0%<br>2: 0.7% / 14.0%<br>3: 2.7% / 19.2%<br>4: 13.3% / 14.0%<br>5: 14.0% / 10.7%<br>6: 18.0% / 4.0%<br>7: 17.3% / 2.0%<br>8: 19.3% / 0.7%<br>9: 8.7% / 0.7%<br>10: 6.0% / 0.0% | n/a |
| | | B530 | BMI (kg/m²): mean (range)<br>39.94 (21.98-71.52) | |
| | | B530 | Waist-to-height ratio: mean (range)<br>0.69 (0.46-0.97) | |
| | | P | Comorbidities: % of population<br>Depressive disorders: 26.0%<br>Eating disorder (especially binge eating disorder): 14.7%<br>Post-traumatic stress disorder: 5.3%<br>Anxiety disorders and panic disorders: 3.3%<br>Pain with somatoform components: 6.0%<br>Other primary diagnosis: 10.6%<br>No mental health disorders according to ICD-10: 49.9% | |
| Fink et al., 2020<br><br>Retrospective cohort<br><br>Germany | N = 45<br><br>Lipoedema group n = 31<br>Age is years: mean (95% CI) 50.6 (47.4-53.7)<br><br>Control group (Lymphoedema) n = 14<br>Age is years: mean (95% CI): 55 (51.5-58.5) | B530 | BMI (kg/m²): mean (95% CI)<br>48.5 (45.5-51.4) | BMI (kg/m²): mean (95% CI)<br>55.2 (51.9-58.5) |
| | | B530 | Waist-to-height ratio: mean (95% CI)<br>0.8 (0.7-0.8) | Waist-to-height ratio: mean (95% CI)<br>0.9 (0.8-0.9) |
| Forner-Cordero et al., 2021<br><br>Cross- | N = 138<br><br>Age in years: median (range) 47.6 (18.0-80.7) | B280<br>B280<br>B28016<br>B28016 | Pain: % of population 92.0%<br>Pain VAS (0-10): mean (95%) 5.7 (5.2-6.3)<br>Knee pain: % of population 63.0%<br>Knee pain VAS (0.10): mean (95% CI) 5.9 (5.5-5.6) | n/a |
| | | B435 | Pitting sign: % of population 14.5% | |

| | | | | |
|---|---|---|---|---|
| sectional study | Age at onset in years: median (range) 14.8 (4.0-60.0) | B435 | Stemmer sign: % of population 13.8% | |
| | | B435 | Lymphangitis attacks: % of population 2.9% | |
| Spain | Lipoedema stage: % of population<br>I: 37.7%<br>II: 34.8% | B530 | BMI ($kg/cm^2$): mean (95% CI)<br>29.9 (28.8-30.9) | |
| | III: 22.5%<br>IV: 5.1% | B530 | Waist-to-hip index: mean (95% CI)<br>0.71 (0.69-0.72) | |
| | Lipoedema type: % of population<br>I: 1.4%<br>II: 13.8%<br>III: 71.0%<br>IV: 11.6%<br>V: 2.2% | B530 | Waist-to-height classification: % of population<br>Slim (0.35-0.41): 10.0%<br>Healthy (0.42-0.48): 31.2%<br>Overweight (0.49-0.53): 19.6%<br>Very Overweight (0.53-0.57): 15.9%<br>Obese (>0.58): 23.2% | |
| | Time elapsed until diagnosis in years: mean (95%CI) 25.5 (25.0-27.9) | B820 | Bruising: % of population: 90.6% | |
| | Positive familiy history: % of population 84.7%<br><br>Triggering factors: % of population<br>Not identified: 20.3%<br>Puberty: 57.2%<br>Pregnancy: 14.5%<br>Menopause: 5.8%<br>Contraceptives: 2.2% | P | Knee osteoarthritis (Kellgren-Lawrence Scale): % of population (n = 63)<br>No radiographic findings of osteoarthritis: 12.7%<br>Minute osteophytes of doubtful clinical significance: 28.6%<br>Definite osteophytes with unimpaired joint space: 25.4%<br>Definite osteophytes with moderate joint space narrowing: 23.8%<br>Definite osteophytes with severe joint space narrowing and subchondral sclerosis: 9.5% | |
| | Upper limbs involvement: % of population 15.2% | P | Comorbidities: % of population<br>Vascular spider: 89.9%<br>Venous insufficiency: 31.2%<br>Othopedic alterations:<br>Normal: 55.1%<br>Cavus feet: 29.7%<br>Flat feet: 15.2%<br>Normal knees: 57.2%<br>Valgus: 39.1%<br>Varus: 3.6% | |
| Gensior et al., 2020 | N = 633 | B280 | Pain max. during (n = 543): % of population<br>Morning: 4.6%<br>Noon: 5.5% | n/a |

| | | | | |
|---|---|---|---|---|
| Cross-sectional study<br><br>Germany | Age in years in categories: % of population<br>15-29: 28.3%<br>30-49: 50.2%<br>50-81: 21.5% | <br><br><br><br>B280<br><br><br><br><br><br><br><br><br><br><br><br><br>B530 | Afternoon: 24.3%<br>Evening: 53.2%<br>Night: 12.3%<br><br>Type of pain (n = 543): % of population<br>Jerking: 11.7%<br>Dull: 24.0%<br>Throbbing: 5.3%<br>Knocking: 1.9%<br>Stinging: 12.1%<br>Pulling: 14.1%<br>Hot: 6.6%<br>Burning: 8.6%<br>Miserable: 11.9%<br>Shivering: 3.9%<br><br>BMI (kg/m$^2$) (n = 541): % of population<br>Normal: 24.0%<br>Pre-obesity: 31.4%<br>Obesity class I: 20.5%<br>Obesity class II: 12.8%<br>Obesity class III: 11.3% | |
| Ghods et al., 2020<br><br>Retrospective cohort<br><br>Germany | N = 106<br><br>Age in years: mean (IQR) 41 (30-51)<br><br>Age at onset: % of population<br>Puberty: 59.0%<br>Pregnancy: 21.0%<br>Contraceptives: 4.0%<br>Menopause: 2.0%<br>Others: 6.0%<br><br>Time until diagnosis in years: median (IQR) 10.0 (5.0-20.0)<br><br>Lipoedema stage: % of population<br>I: 45.5%<br>II: 55.7%<br>III: 76.5% | B280<br><br>B530<br><br><br>P | Pain VAS (0-100): median 80<br><br>BMI (kg/m$^2$): median (IQR)<br>31.6 (26.4-38.8)<br><br>Comorbidities: % of population<br>Obesity: 37.6%<br>Allergies: 36.8%<br>Hypothyroidism: 31.1%<br>Sleep disorders: 25.5%<br>Depression: 25.5%<br>Hypertension: 24.5%<br>Migraine: 22.6%<br>Skin disorders: 18.9%<br>Asthma: 17.9%<br>Gastrointesial disorders: 10.4%<br>Rheumatism: 8.5%<br>Dyslipidemia: 6.6% | n/a |

| | | | | |
|---|---|---|---|---|
| | Upper limbs involvement: % of population 61.3%<br><br>Positive family history: % of population 73.0%<br>Mother: 38.0%<br>Grandmother: 17.0%<br>Aunt: 8.0%<br>Sister: 6.0%<br>Cousin: 5.0% | | Diabetes type II: 4.7%<br>PCO syndrome: 2.8% | |
| Gould et al., 2019<br><br>Cross-sectional study<br><br>USA | N = 19<br><br>Age in years: mean ± sd<br>54.8 ± 12.1<br><br>Lipoedema stage: % of population<br>I: 26.3%<br>II: 21.1%<br>III: 21.1%<br>IV: 31.6% | B530<br><br>B435 | BMI (kg/m²): mean ± sd<br>35.9 ± 10.7<br><br>Lymphatic function: % of legs<br>*Method: Lymphoscintigraphy*<br>Delayed uptake with notable retention of radiotracer in the feet: 36.8%<br>Collateral lymphatic vessels: 34.2%<br>Some degree of dermal backflow: 21.1%<br>Multiple inguinal lymph nodes visualized: 94.7%<br>Para iliac lymph node visualization: 92.1%<br>Hepatic activity: 84.2%<br>Nonpathologic lymphatic transport: 36.8%<br>Pathologic lymphatic transport: 63.2%<br>Transport index: mean ± sd 12.5 ± 8.39 | n/a |
| Hamatschek et al., 2022<br><br>Cross-sectional study<br><br>Germany | N = 511<br><br>Age in years: mean ± sd 40.16 ± 12.45<br><br>Age at onset in years: mean ± sd 19.66 ± 10.00<br><br>Age at diagnosis in years: mean ± sd 36.69 ± 11.79<br><br>Time between onset and diagnosis in years: mean ± sd 16.11 ± 11.39<br><br>Lipoedema stage: % of population<br>Legs<br>I: 8.6% | B152<br><br>B270<br><br>B280<br>B28012<br>B28014<br>B28015<br>B28015<br>B28015<br><br>B435<br><br>B530<br><br>B780 | PHQ-9 (0-27) (n = 337): mean ± sd 10.84 ±6.39<br><br>Hypersensitivity to touch VAS (0-10): mean ± sd 7.32 ± 2.42<br><br>Pain in affected areas VAS (0-10): mean ± sd 6.68 ± 2.32<br>Pain in belly VAS (0-10): mean ± sd 4.00 ± 2.72<br>Pain in arms VAS (0-10): mean ± sd 5.33 ± 2.72<br>Pain in thighs VAS (0-10): mean ± sd 6.34 ± 2.5<br>Pain in lower legs VAS (0-10): mean ± sd 6.18 ± 2.53<br>Pain in buttock VAS (0-10): mean ± sd 4.65 ± 2.62<br><br>Swelling VAS (0-10): mean ± sd 7.04 ± 2.41<br><br>BMI (kg/m²): mean ± sd 33.13 ± 7.8<br><br>Feeling of heavy legs VAS (0-10): mean ± sd 8.21 ± 1.95 | n/b |

| | | | |
|---|---|---|---|
| | II: 57.8%<br>III: 33.6% | B780<br>B780<br>B780 | Feeling of tired legs VAS (0-10): mean ± sd 7.79 ± 2.17<br>Muscle cramps VAS(0-10): mean ± sd 4.63 ± 2.86<br>Feeling of tension in the legs VAS (0-10): mean ± sd 7.49 ± 2.19 |
| | Arms:<br>I: 11.8%<br>II: 36.0% | B820 | Bruising (hematomas) VAS (0-10): mean ± sd 7.63 ± 2.31 |
| | III: 9.9%<br>0: 42.3% | B840 | Itching VAS (0-10): mean ± sd 4.78 ± 3.00 |
| | Positive family history: % of population<br>Mother 33.6% | D450 | Impairment in walking VAS (0-10): mean ± sd 6.45 ± 2.78 |
| | Grandmother (in general) 28.4%<br>Grandmother maternal 15.4% | S810 | Skin complications VAS (0-10): mean ± sd 4.11 ± 2.98 |
| | Sister 14.6%<br>Grandmother paternal 10.3%<br>Aunt paternal 9.8%<br>Aunt maternal 9.2%<br>Daughter 2.3% | P | WHOQOL-BREF (n=353): mean ± sd<br>Mean of all the domains: 60.5 ± 16.02<br>Physical 54.54 ± 20.1<br>Mental 51.91 ± 18.67<br>Environment 71.85 ± 16.00 |
| | | P | Smoking behavior: % of population<br>Nonsmoker: 87.2%<br><5 per day: 6.4%<br>5–10 per day: 3.4%<br>11–15 per day: 1.8%<br>16–20 per day:1.2% |
| | | P | Comorbidities: % of population<br>Hypothyroidism 31.3%<br>Joint pain 27.3%<br>Skin problems 19.2%<br>Hypertonia 18.5%<br>Thrombosis 9.3%<br>Type II diabetes 3.0% |
| Harwood et al., 1996 | N = 10 | B270 | Tenderness: % of population 50.0% |
| Case series | Age in years: mean (range)<br>44.6 (25-79) | B280 | Pain: % of population 30.0% |
| UK | Age at onset: % of population<br>Puberty: 60.0% | B415 | Venous function: % of population<br>*Method: Photoplethysmography*<br>Normal*: right leg 70.0% /left leg 80.0%<br>* *(Normal refilling time >40 seconds)* |

| | | | | |
|---|---|---|---|---|
| | Positive family history: % of population<br>40.0% | B435 | Lymphatic function: % of population<br>*Method: Quantitative lymphoscintigraphy*<br>Normal*: right leg 60.0%/left leg 80.0%<br>* *(Normal uptake at 120 minutes > 8%)* | |
| | | B530 | BMI ($kg/m^2$): % of population<br>Obesity: 40.0% | |
| | | B820 | Easy bruising: % of population 40.0% | |
| | | P | Superficial varicosities: % of population 20.0% | |
| Herbst et al., 2015 | N = 51 | B134 | Difficulty sleeping: % of population ± se<br>Stage I: 80.0 ± 22.0 / stage II: 64.3 ± 12.0 /stage III: 45.5 ± 23.0 | n/a |
| Cross-sectional study | Age in years: mean ± sem 50±13<br>F/M (%): 98.0%/ 2.0% | B140 | Poor concentration: % of population ± se<br>Stage I: 100.0 / stage II: 52.0 ± 10.0 / stage III: 40.0 ± 26.0 | |
| USA | Lipoedema stage: % of population<br>I: 8.6%<br>II: 56.9% | B265 | Numbness: % of population ± se<br>Stage I: 0.0 / stage II: 43.0 ± 14.0/ stage III: 82.0 ± 14.0 | |
| | III: 22.4%<br>IV: 12.1%<br>Age at onset in years (n= 41): mean ± sem<br>24.0 ± 13.0 | B280 | Pain (n = 39): % of population<br>No pain: 10.3%<br>Daily pain: 89.7% | |
| | | B280 | Burning pain: % of population ± se<br>Stage I: 0 / stage II: 40.0 ± 14.0 / stage III: 25.0 ± 26.0 | |
| | | | Location of pain: % of population ± se | |
| | | B280 | Pain in the eyes: 14 ± 0.4% | |
| | | B280 | Pain in the fat: 49 ± 0.5% | |
| | | B280 | Pain in the muscle: 37 ± 0.5% | |
| | | B28010 | Pain in the head 25 ± 0.4% | |
| | | B28011 | Pain in the ribs: 14 ± 0.4% | |
| | | B28012 | Pain in the stomach 16 ± 0.4% | |
| | | B28013 | Pain in the back: 44 ± 0.5% | |
| | | B28014 | Pain in the arm 25 ± 0.4% | |
| | | B28015 | Pain in the legs 39 ± 0.5% / Pain in the feet 23 ± 0.4% | |
| | | B28016 | Pain in the joints 57 ± 0.5% | |
| | | B430 | Blood clot: % of population ± se<br>Stage I: 0 / stage II: 0 / stage III: 16.7 ± 28.0† | |

| | |
|---|---|
| B435 | Edema: % of population ± se<br>Stage I: 50.0 ± 41.0 /stage II: 74.0 ± 10.0 /stage III: 100.0† |
| B4552 | Fatigue: % of population ± se<br>Stage I: 75.0 ± 29.0/ stage II: 77.0 ± 9.0 / stage III: 91.7 ± 8.7 |
| B460 | Palpitations: % of population ± se<br>Stage I: 0 / stage II: 35.0 ± 15.0 / stage III: 50.0 ± 21.0 |
| B460 | Shortness of breath: % of population ± se<br>Stage I 0 / stage II: 39.3 ± 15.0 / stage III: 50.0 ± 21.0 |
| B525 | Constipation: % of population ± se<br>Stage I: 25.0 ± 0.5 / stage II 35.0 ± 10.0 / stage III 45.0 ± 23.0 |
| B530 | BMI (kg/m$^2$): mean ± sem 38.0 ± 12.0 |
| B535 | Early satiety: % of population ± se<br>Stage I: 50.0 ± 41.0 / stage II: 36.7 ± 15.0 / stage III: 18.2 ± 27.0 |
| B535 | Nausea: % of population ± se<br>Stage I: 0 / stage II: 29.0 ± 15.0 / stage III: 33.3 ± 25.0 |
| B535 | Bloating: % of population ± se<br>Stage I: 75.0 ± 29.0 / stage II: 58.0 ± 12.0 / stage III: 45.5 ± 23.0 |
| B550 | Body temperature: % of population ± se<br>High: stage I: 0 / stage II: 20.0 ± 17.0 / stage III: 45.0 ± 23.0<br>Low: stage I: 75.0 ± 29.0 / stage II: 41.4 ± 14.0 / stage III: 33.3 ± 25.0 |
| B620 | Nocturia: % of population ± se<br>Stage I: 75.0 ± 29.0 / stage II: 55.0 ± 12.0 / stage III: 66.7 ± 17.0 |
| B620 | Frequent urination: % of population ± se<br>Stage I: 25.0 ± 50.0 / stage II: 41.9±14.0 / stage III: 66.7±17.0 |
| B710 | Beighton score (0-9): mean ± se 4.2 ± 0.5 |
| B730 | Muscle weakness: % of population ± se |

| | | | | |
|---|---|---|---|---|
| | | | Stage I: 25.0 ± 50.0 /stage II: 54.8 ± 12.0/ stage III: 50.0 ± 24.0 | |
| | | B820 | Easy bruising: % of population ± se<br>Stage I: 75.0 ± 29.0 / stage II: 68.0 ± 10.4 /stage III: 83.0 ± 12.0 | |
| | | B840 | Itching: % of population ± se<br>Stage I: 75.0 ± 29.0 / stage II: 39.0 ± 14.3 /stage III: 58.0 ± 20.0 | |
| | | B850 | Hair loss: % of population ± se<br>Stage I: 0 / stage II: 48.0 ± 15.0 / stage III: 70.0 ± 18.0 | |
| | | D240 | Stress: % of population ± se<br>Stage I: 69.0 ± 11.0 / stage II: 83.0 ± 12.0 / stage III: 83.0 ± 12.0 | |
| | | P | Comorbities: % of population ± se<br>Abdominal or pelvic surgery: 48.0 ± 0.5<br>Arthritis: 38.0 ± 0.5<br>Dyslipidemia: 38.0 ± 0.5<br>Hypertension: 31.0 ± 0.5<br>Type 2 diabetes: 2.0 ± 0.1<br>Visible veins on legs:<br>Stage I: 100 / stage II: 72.0 ± 10.0 / stage III: 27.3 ± 27.0 | |
| Hirsch et al., 2018<br><br>Cross-sectional study<br><br>Germany | N = 244<br><br>Lipoedema group n = 136<br>Age in years: mean ± sd 39.27 ± 12.65<br><br>Control group 1 (Lipohypertrophy) n = 42<br>Age in years: mean ± sd 42.94 ± 12.22<br><br>Control group 2 (Obesity) n = 30<br>Age in years: mean ± sd 41.44 ± 11.67<br><br>Control group 3 (healthy) n = 36<br>Age in years: mean ± sd 50.88 ± 17.73 | B530 | BMI (kg/m²): mean ± sd<br>29.83 ± 6.75 | BMI (kg/m²): mean ± sd<br>Control group 1: 30.74 ± 7.49<br>Control group 2: 46.04 ± 10.08<br>Control group 3: 22.4 ± 3.24 |
| Iker et al., 2018<br><br>Cross-sectional study | N = 12<br><br>Lipoedema group n = 12<br>Age in years: mean (range)<br>41.8 (range 24.0-69.0) | B530<br><br><br><br>S810 | BMI (kg/m²): mean (range)<br>24.6 (20.18-32.0)<br><br><br>Cutis thickness (cm): mean (range)<br>*Method: Ultrasound* | BMI (kg/m²): mean (range)<br>Control group 1: 24.2 (17.9-33.85)<br>Control group 2: 23.3 (20.2-29.55)<br><br>Cutis thickness: (cm) mean (range)<br>*Method: Ultrasound* |

| | | | | |
|---|---|---|---|---|
| USA | Control group 1 (Lymphoedema) n = 10<br>Age in years: mean (range) 63.1 (21.0-83.0)<br><br>Control group 2 (healthy) n = 8<br>Age in years: mean (range) 58.1 (21.0-80.0) | | Ankle: 0.152 cm (range 0.1-0.2)<br>Calf: 0.145 cm (range 0.1-0.2)<br>Thigh: 0.213 cm (range 0.1-0.7) | Control group 1:<br>Ankle: 0.249 cm (range 0.18-0.4)<br>Calf: 0.268 cm (range 0.18-0.35)<br>Thigh: 0.242 cm (range 0.1-0.35)<br>Control group 2:<br>Ankle: 0.165 cm (range 0.1-0.2)<br>Calf: 0.155 cm (range 0.1-0.2)<br>Thigh: 0.168 cm (range 0.1-0.2) |
| Jeziorek et al., 2022<br><br>Controlled clinical trial<br><br>Poland | N = 91<br><br>Lipoedema group 1 n = 46<br>Age in years: mean ± sd 42.5 ± 13.4<br><br>Lipoedema group 2 n = 45<br>Age in years: mean ± sd 44.0 ± 12.3<br><br>Lipoedema stage: % of total population<br>I: 44.0%<br>II: 41.8%<br>III: 13.2%<br>IV: 1.1% | B530<br><br><br><br>B530<br><br><br><br><br><br><br><br><br><br>B540 | BMI (kg/m²): mean ± sd<br>LG 1: 31.0 ± 6.8<br>LG 2: 33.5 ± 9.2<br><br>Body composition: mean ± sd LG 1/ LG 2<br>*Method: Bioelectrical impedance analysis*<br>Lean body mass (kg): 52.7 ± 7.7 / 54.5 ± 9.5<br>Percentage of body fat (%): 37.4 ± 7.0 / 38.7 ± 6.9<br>Mass body fat (kg): 33.2 ± 12.3 / 36.7 ± 14.7<br>Visceral fat level: 13.3 ± 5.1 / 14.2 ± 4.9<br>Mass body fat left leg (kg): 6.1 ± 2.2 / 6.7 ± 2.6<br>Mass body fat right leg (kg) 6.1 ± 2.2 / 6.6 ± 2.6<br><br>RMR (kcal/day): mean ± sd<br>*Method: Indirect calorimetry*<br>LG 1: 1675.0 ± 306<br>LG 2: 1749.0 ± 329 | n/a |
| Kruppa et al., 2020<br><br>Retrospective cohort<br><br>Germany | N = 106<br><br>Age in years: median (IQR): 41 (30-51)<br><br>Lipoedema stage: % of population<br>I: 10.4%<br>II: 57.6%<br>III: 32.1%<br><br>Upper limbs involvement: % of population 61.3%<br><br>Positive family history: % of population 72.6%<br><br>Triggering factors: % of population | B180<br><br>B270<br><br>B280<br><br>B780<br><br>B530<br><br>B820<br><br>B640 | Body image impairment VAS (0-100): median (IQR) 90 (80-100)<br><br>Sensitivity to pressure VAS (0-100): median (IQR) 80 (70-90)<br><br>Pain VAS (0-100): median (IQR) 80 (70-90)<br><br>Feeling of tension VAS (0-100): median (IQR) 80 (60-90)<br><br>BMI (kg/m²): (median (IQR) 31.6 (26.4-38.8)<br><br>Bruising VAS (0-100): median (IQR) 80 (60-90)<br><br>Impairment of quality of sexual life VAS (0-100): median (IQR) 75 (40-82.5) | n/a |

| | | | | |
|---|---|---|---|---|
| | Puberty: 58.5%<br>Pregnancy: 20.8% | P | General impairment VAS (0-100): median (IQR) 90 (80-100) | |
| | Contraceptives: 3.8%<br>Menopause: 1.9%<br>Other: 5.7% | P | Comorbidities: % of population<br>Obesity: 37.6%<br>Allergies: 36.85%<br>Hypothyroidism: 31.1%<br>Depression: 25.5%<br>Sleep disorders: 25.5%<br>Hypertenion: 24.5%<br>Migriane: 22.6%<br>Skin disorders: 18.9%<br>Asthma: 17.9%<br>Gastrointestinal disorders: 10.4%<br>Rheumatism: 8.5%<br>High cholesterol: 6.6%<br>Diabetes type II: 4.7%<br>PCO syndrome: 2.8%<br>Diabetes type I: 0.0% | |
| Lohrman et al., 2009<br><br>Cross-sectional study<br><br>Germany | N = 13<br><br>Lipoedema group n = 5<br>Age: not addressed<br>Control group (Lipolymphoedema) n = 8<br>Age: not addressed | B435 | Lymphatic function: % of legs<br>*Method: Magnetic Resonance Lymphangiography*<br>High signal intensity areas indicating lymphoedema: 0.0%<br>Dermal back-flow area with collateral lymphatic vessels: 0.0%<br>Concomitant venous enhancement: 100.0%<br><br>Time of highest contrast enhancement Lymphatic vessels lower leg: % of legs<br>15 min 0.0%<br>25 min 20.0%<br>35 min 50.0%<br>45 min 30.0%<br>55 min 0.0%<br>Lymphatic vessels upper leg: % of legs<br>15 min 0.0%<br>25 min 0.0%<br>35 min 40.0%<br>45 min 40.0%<br>55 min 20.0%<br>Inguinal lymph node areas: % of legs<br>15 min 0.0% | Lymphatic function: % of legs<br>*Method: Magnetic Resonance Lymphangiography*<br>High signal intensity areas indicating lymphoedema: 100.0%<br>Dermal back-flow area with collateral lymphatic vessels: 0.0%<br>Concomitant venous enhancement: 100.0%<br><br>Time of highest contrast enhancement: % of legs Lymphatic vessels lower leg: % of legs<br>15 min 0.0%<br>25 min 0.0%<br>35 min 38.0%<br>45 min 44.0%<br>55 min 18.0%<br>Lymphatic vessels upper leg: % of legs<br>15 min 0.0%<br>25 min 0.0%<br>35 min 13.0%<br>45 min 57.0%<br>55 min 30.0%<br>Inguinal lymph node areas: % of legs<br>15 min 0.0% |

| | | | | |
|---|---|---|---|---|
| | | | 25 min 0.0%<br>35 min 30.0%<br>45 min 50.0%<br>55 min 20.0% | 25 min 0.0%<br>35 min 6.0%<br>45 min 20.0%<br>55 min 44.0% |
| | | S420 | Lymphatic anatomy: % of legs<br>*Method: Magnetic Resonance Lymphangiography*<br>Enlarged lymphatic vessels lower leg: 40.0%<br>Enlarged lymphatic vessel upper leg: 20.0% | Lymphatic anatomy: % of legs<br>*Method: Magnetic Resonance Lymphangiography*<br>Enlarged lymphatic vessels lower leg: 50.0%<br>Enlarged lymphatic vessel upper leg: 19.0% |
| Marshall et al., 2011<br><br>Cross-sectional study<br><br>Germany | N = 62<br><br>Lipoedema group n= 24<br>Age in years: mean ± sd 40.67 ± 11.68<br><br>Control group (non-Lipoedema group) n = 38<br>Age in years: mean ± sd 45.84 ± 12.92 | B530 | BMI (kg/m$^2$): mean ± sd<br>27.65 ± 3.69 | BMI (kg/m$^2$): mean ± sd<br>23.5 ± 2.74 |
| | | S810 | Thickness cutis (mm): mean ± sd<br>*Method: Ultrasound*<br>Left leg proximal to the ankle, medial side: 1.89 ± 0.28<br>Right leg proximal to the ankle, medial side: 1.76 ± 0.28 | Thickness cutis (mm): mean ± sd<br>*Method: Ultrasound*<br>Left leg proximal to the ankle, medial side: 1.62 ± 0.39<br>Right leg proximal to the ankle, medial side: 1.65 ± 0.41 |
| | | S810 | Thickness subcutis (mm): mean ± sd<br>*Method: Ultrasound*<br>Left leg proximal to the ankle, medial side: 16.5 ± 4.08<br>Right leg proximal to the ankle, medial side: 14.6 ± 3.86 | Thickness subcutis (mm): mean ± sd<br>*Method: Ultrasound*<br>Left leg proximal to the ankle, medial side: 11.2 ± 2.85<br>Right leg proximal to the ankle, medial side: 10.6 ± 2.16 |
| Melander et al., 2021<br><br>Qualitative study<br><br>Sweden | N = 14<br><br>Age in years: median (range) 46.4 (30.0-60.0)<br><br>Time since diagnosis in years: median (range) 4.3 (1-15) | B152 | 'The women blamed themselves and felt guilty while striving to take responsibility, and they felt alone and left behind without any help to manage daily living.' | n/a |
| | | E355 | 'They lacked professional care by receiving unsupportive advice on how to manage. A striking issue that we aim to highlight regarding the results of this study are the fact that women with lipoedema are treated unjustly in healthcare encounters.' | |
| | | E460 | 'In social settings, as well as in healthcare encounters, women with lipoedema were fat-shamed and viewed by others as a person who lacks character.' | |
| Münch, 2017<br><br>Prospective cohort<br><br>Switzerland | N = 141<br><br>Age in years: mean ± sd 37.2 ± 9.68 | B180 | Disturbing body proportions VAS (0-10): mean 8.5 | n/a |
| | | B280 | Pain VAS (0-10): mean 6 | |
| | | B270 | Sensitivity to pressure VAS (0-10): mean 5.8 | |
| | | B530 | BMI (kg/m$^2$): mean ± sd 26.6 ± 5.14 | |

| | | | | |
|---|---|---|---|---|
| | | B820 | Easy bruising VAS (0-10): mean 5.9 | |
| | | D450 | Restrictions when walking VAS (0-10): mean 3.7 | |
| | | P | Limited quality of life VAS (0-10): mean: 6.3 | |
| Nemes et al., 2018<br><br>Cross-sectional study<br><br>Hungary | N = 105<br><br>Lipoedema group n = 25<br>Age in years: mean ± sd 42.5 ± 12.2<br>F/M (%): 100%/ 0%<br><br>Control group 1 (Lymphoedema) n = 26<br>Age in years: mean ± sd 46.5 ± 11.5<br>F/M (%): 92.3%/ 7.7%<br><br>Control group 2 (healthy) n = 54<br>Age in years: mean ± sd 40.7 ± 14.0<br>F/M (%): 94.4%/ 5.6% | B410 | Left ventricular (LV) functional parameters: mean ± sd<br>*Method: Two-dimensional echocardiography*<br>LV ejection fraction: % ± sd 67.5 ± 3.5<br>e (cm/s): 87.3 ± 18.6<br>a (cm/s): 78.1 ± 17.8<br>e/a- ratio: 1.18 ± 0.40 | Left ventricular (LV) functional parameters: mean ± sd CG 1/CG2<br>*Method: Two-dimensional echocardiography*<br>LV ejection fraction: % ± sd 69.7 ± 4.8<br>e (cm/s): 76.8 ± 15.1 / 78.3±18.1<br>a (cm/s): 67.2 ± 14.6 / 65.5±17.6<br>e/a- ratio: 1.21 ± 0.36 / 1.29±0.37 |
| | | B410 | LV functional parameters (n = 19): mean ± sd<br>*Method: Three-dimensional speckle-tracking echocardiography*<br>Time-to-peak: msec ± sd<br>LV basal rotation: 392 ± 167<br>LV apical rotation: 335 ± 86<br>LV twist: 308 ± 60 | LV functional parameters (n = 19): mean ± sd CG 1/CG2<br>*Method: Three-dimensional speckle-tracking echocardiography*<br>Time-to-peak: msec ± sd<br>LV basal rotation: 350 ± 134 / 361 ± 105<br>LV apical rotation: 330 ± 56 / 343 ± 105<br>LV twist: 321 ± 70 / 354 ± 98 |
| | | S410 | LV anatomical parameters: mean ± sd<br>*Method: Two-dimensional echocardiography*<br>Left atrial diameter (mm): 39.9 ± 4.4<br>LV end-diastolic diameter (mm): 50.3 ± 3.3<br>LV end-diastolic volume (ml): 121.4 ± 18.3<br>LV end-systolic diameter (mm): 31.4 ± 2.7<br>LV end-systolic volume (ml): 40.0 ± 8.2<br>Interventricular septum (mm): 8.6 ± 0.9†<br>LV posterior wall (mm): 8.6 ± 0.9 | LV anatomical parameters: mean ± sd CG 1/CG 2<br>*Method: Two-dimensional echocardiography*<br>Left atrial diameter (mm): 37.7 ± 4.3 / 35.4±4.1<br>LV end-diastolic diameter (mm): 47.8 ± 4.0 /46.9±3.6<br>LV end-diastolic volume (ml): 109.3 ± 20.3 / 98.4±21.5<br>LV end-systolic diameter (mm): 29.1 ± 3.5 / 36.0±18.7<br>LV end-systolic volume (ml): 33.7 ± 9.6 / 33.8±8.1<br>Interventricular septum (mm): 8.0 ± 0.9 / 8.8±1.5<br>LV posterior wall (mm): 8.2 ± 1.1 / 9.0±1.7 |
| | | S410 | LV anatomical parameters (n = 19): mean ± sd<br>*Method: Three-dimensional speckle-tracking echocardiography*<br>LV basal rotation: degree ± sd -3.75 ± 2.01<br>LV apical rotation: degree ± sd 6.40 ± 2.63<br>LV twist: degree ± sd 10.04 ± 3.56 | LV anatomical parameters (n = 19): mean ± sd<br>*Method: Three-dimensional speckle-tracking echocardiography*<br>LV basal rotation: degree ± sd -3.17 ± 1.50 / -4.22 ± 2.17<br>LV apical rotation: degree ± sd 10.51 ± 4.20 / 9.61 ± 4.25<br>LV twist: degree ± sd 13.68 ± 4.69 / 13.83 ± 4.89 |
| | | B530 | BMI (kg/m$^2$): mean ± sd<br>29.9 ± 2.79 | BMI (kg/m$^2$): mean ± sd CG 1/CG 2<br>Control group 1: 27.64 ± 2.6/ n/a |
| | | P | Comorbidities: % of population<br>Hypertension: 0.0% | Comorbidities CG 1/CG 2: % of population<br>Hypertension: 4.0% / 0.0% |

| | | | | |
|---|---|---|---|---|
| | | | Diabetes mellitus 0.0%<br>Hyperlipidaemia 0.0% | Diabetes mellitus 0.0% / 0.0%<br>Hyperlipidaemia 4.0% / 0.0% |
| Nemes et al., 2019<br><br>Cross-sectional study and prospective cohort<br><br>Hungary | N = 72<br><br>Lipoedema group n = 24<br>Age in years: mean ± sd 43.4 ± 11.7<br><br>Control group (healthy) n = 48<br>Age in years: mean ± sd 41.0 ± 14.0 | B410 | Mitral annular functional parameters: mean ± sd<br>*Method: Three-dimensional speckle-tracking echocardiography*<br>Mitral annular fractional area change (%): 47.78 ± 18.18<br>Mitral annular fractional shortening (%): 30.16 ± 14.07 | Mitral annular functional parameters: mean ± sd<br>*Method: Three-dimensional speckle-tracking echocardiography*<br>Mitral annular fractional area change (%): 55.39 ± 14.01<br>Mitral annular fractional shortening (%): 34.57 ± 15.01 |
| | | S410 | Mitral annular anatomical parameters: mean ± sd<br>*Method: Three-dimensional speckle-tracking echocardiography*<br>End-diastolic mitral annular diameter (cm): 2.54 ± 0.51<br>End-diastolic mitral annular area ($cm^2$): 8.45 ± 2.43<br>End-diastolic mitral annular perimeter (cm): 10.92 ± 1.46<br>End-systolic mitral annular diameter (cm): 1.75 ± 0.46<br>End-systolic mitral annular area ($cm^2$): 4.30 ± 1.72<br>End-systolic mitral annular perimeter (cm): 7.84 ± 1.40 | Mitral annular anatomical parameters: mean ± sd<br>*Method: Three-dimensional speckle-tracking echocardiography*<br>End-diastolic mitral annular diameter (cm): 2.42 ± 0.39<br>End-diastolic mitral annular area ($cm^2$): 7.33 ± 2.21 End-diastolic mitral annular perimeter (cm): 10.17 ± 1.54<br>End-systolic mitral annular diameter (cm): 1.55 ± 0.39<br>End-systolic mitral annular area ($cm^2$): 3.21 ± 1.12<br>End-systolic mitral annular perimeter (cm): 6.75 ± 1.10 |
| | | P | Comorbidities: % of population<br>Hypertension: 0.0%<br>Diabetes mellitus 0.0%<br>Hyperlipidaemia 0.0% | Comorbidities: % of population<br>Hypertension: 0.0%<br>Diabetes mellitus 0.0%<br>Hyperlipidaemia 0.0% |
| Nemes et al., 2020<br><br>Cross-sectional study and prospective cohort<br><br>Hungary | N = 47<br><br>Lipoedema group n = 19<br>Age in years: mean ± sd 42.2 ± 12.4<br><br>Control group (healthy) n = 28<br>Age in years: mean ± sd 42.0± 9.8 | B410 | Left ventricular (LV) functional parameters (%): mean ± sd<br>*Method: Three-dimensional speckle-tracking echocardiography*<br>Ejection fraction: 61.1 ± 5.5<br><br>LV global strains (%): mean ± sd<br>Radial: 28.3 ± 11.1<br>Circumferential: −30.2 ± 4.0<br>Longitudinal: −18.4 ± 2.6<br>3D: 30.3 ± 10.9<br>Area: −43.1 ± 4.7<br><br>LV segmental strains (%): mean ± sd<br>Radial: 31.1 ± 10.9<br>Circumferential: −30.9 ± 4.3<br>Longitudinal: −19.2 ± 2.7<br>3D: 33.0 ± 10.9<br>Area: −44.2 ± 4.9<br>RSbasal: 35.9 ± 16.7<br>RSmid: 35.8 ± 11.6<br>RSapex: 16.7 ± 8.3<br>CSbasal: −27.7 ± 8.0 | Left ventricular (LV) functional parameters (%): mean ± sd<br>*Method: Three-dimensional speckle-tracking echocardiography*<br>Ejection fraction: 56.3 ± 6.5<br><br>LV global strains (%): mean ± sd<br>Radial: 24.3 ± 10.5<br>Circumferential: −25.9 ± 5.4<br>Longitudinal: −17.2 ± 2.3<br>3D: 26.6 ± 10.3<br>Area: −39.7 ± 5.2<br><br>LV segmental strains (%): mean ± sd<br>Radial: 26.8 ± 9.8<br>Circumferential: −27.0 ± 5.4<br>Longitudinal: −17.9 ± 2.2<br>3D: 29.0 ± 9.7<br>Area: −40.8 ± 5.1<br>RSbasal: 33.1 ± 15.5<br>RSmid: 27.4 ± 10.2<br>RSapex: 16.6 ± 7.7<br>CSbasal: −24.4 ± 5.6 |

| | | | | |
|---|---|---|---|---|
| | | | CSmid: −32.7 ± 8.0<br>CSapex: −33.2 ± 8.0<br>LSbasal: −19.9 ± 5.0<br>LSmid: −18.0 ± 3.3<br>LSapex: −20.0 ± 4.9<br>3DSbasal: 38.3 ± 17.4<br>3DSmid : 37.2 ± 11.1<br>3DSapex: 18.6 ± 9.0<br>ASbasal: −41.3 ± 8.9<br>ASmid: −44.8 ± 4.6<br>ASapex: −47.6 ± 9.4 | CSmid: −26.7 ± 6.1<br>CSapex: −31.6 ± 9.8<br>LSbasal: −22.1 ± 4.6<br>LSmid: −14.4 ± 2.8<br>LSapex: −16.9 ± 5.3<br>3DSbasal: 35.8 ± 14.9<br>3DSmid: 28.8 ± 10.3<br>3DSapex: 18.2 ± 7.9<br>ASbasal: −40.4 ± 6.6<br>ASmid: −38.0 ± 6.4<br>ASapex: −44.4 ± 10.7 |
| | | B530 | BMI ($kg/m^2$): mean ± sd<br>34.1 ± 7.0 | BMI ($kg/m^2$): mean ± sd<br>23.3 ± 4.7 |
| | | S410 | Left ventricular (LV) anatomical parameters: mean ± sd<br>*Method: Two-dimensional echocardiography*<br>LV end-diastolic volume (ml): 86.6 ± 17.3<br>LV end-systolic volume (ml): 33.4 ± 6.9 | Left ventricular (LV) anatomical parameters: mean ± sd<br>*Method: Two-dimensional echocardiography*<br>LV end-diastolic volume (ml): 74.0 ± 13.3<br>LV end-systolic volume (ml): 32.1 ± 6.6 |
| Rapprich et al., 2011<br><br>Prospective cohort<br><br>Germany | N = 25<br><br>Age in years: mean ± sd 38.0 ± 12.5<br><br>Lipoedema location: % of population<br>Whole leg: 80.0%<br>Thigh: 12.0%<br>Lower leg: 8.0% | B270 | Sensitive to touch VAS (0-10): mean 6.4 | n/a |
| | | B280 | Pain VAS (0-10): mean ± sd 7.2 ± 2.2 | |
| | | B435 | Swelling VAS (0-10): mean 6.9 | |
| | | B780 | Tension in legs VAS (0-10): mean 7.7 | |
| | | B780 | Muscle cramps VAS (0-10): mean 2.7 | |
| | | B780 | Heavy legs VAS (0-10): mean 8.4 | |
| | | B780 | Tired legs VAS (0-10): mean 8.4 | |
| | | B820 | Bruise easily VAS (0-10): mean 7.9 | |
| | | B840 | Itching VAS (0-10): mean 4.2 | |
| | | S810 | Skin involvement VAS (0-10): mean 3.5 | |
| | | D450 | Difficulty walking VAS (0-10): mean 4.6 | |

| | | | | |
|---|---|---|---|---|
| | | E580 | Received manual lymph drainage: % of population 60.0% | |
| | | P | Quality of life VAS (0-10): mean ± sd 8.7 ± 1.7 | |
| Rasmussen et al., 2022<br><br>Cross-sectional study<br><br>USA | N = 29<br><br>Lipoedema group n = 20<br>Age in years: mean (range) 39.0 (23-48)<br>F/M (%): 100.0%/ 0.0%<br><br>Lipoedema stage: % of population<br>I: 40.0%<br>II: 55.0%<br>III: 5.0%<br><br>Control group n = 9<br>F/M (%): 33.3%/66.7<br>Age in years: mean (range) 44.9 (30-58) | B435 | Lymphatic function: % of population<br>*Method: Near-infrared fluorescence lymphatic imaging*<br>Legs with stage I disease (n = 16):<br>Interstitial backflow: 56.0%<br>Exhibited distinct fluorescent signal attentuation: 81.0%<br>Legs with stage II disease (n = 22):<br>Interstitial backflow: 36.0%<br>Exhibited distinct fluorescent signal attentuation: 82.0%<br><br>Arms (n = 543):<br>Interstitial backflow: 5.0%<br>Lymphatic propulsion rates (events/min): mean ± sd<br>Stage I: 1.4 ± 0.6<br>Stage II: 1.4 ± 0.7<br>Stage III: 1.8 ± 0.1 | Lymphatic function: % of population<br>*Method: Near-infrared fluorescence lymphatic imaging*<br>Interstitial backflow: 5.6%<br>Exhibited distinct fluorescent signal attenuation: 5.6%<br>Lymphatic propulsion rates (events/min): mean ± sd<br>0.9 ± 0.4 |
| | | S420 | Lymphatic anatomy: % of population<br>*Method: Near-infrared fluorescence lymphatic imaging*<br>Legs with stage I disease (n = 16):<br>Evidence of segmentation/varicosity: 56.0%<br>Tortuous vessels: 25.0%<br>Dilated vessels: 94.0%<br>Radiating vessels: 25.0%<br>Diffuse lymphatic structures: 19.0%<br><br>Legs with stage II disease (n = 22):<br>Evidence of segmentation/varicosity: 50.0%<br>Tortuous vessels: 36.0%<br>Radiating vessels: 68.0%<br>Diffuse lymphatic structures: 23.0%<br><br>Arms (n = 40):<br>Evidence of segmentation/varicosity: 7.5%<br>Tortuous vessels: 5.0%<br>Dilated vessels: 2.5% | Lymphatic anatomy: % of population<br>*Method: Near-infrared fluorescence lymphatic imaging*<br>Evidence of segmentation: 11.1%<br>Tortuous vessels: 5.6%<br>Dilated vessels: 0.0%<br>Radiating vessels: 22.2%<br>Diffuse lymphatic structures: 19.0% |
| | | B530 | BMI (kg/m$^2$): mean (range)<br>28.8 (21.4-36.0) | BMI (kg/m$^2$): mean (range)<br>29.6 (23.5-37.6) |

| | | | | |
|---|---|---|---|---|
| Rockson et al., 2022<br><br>Retrospective cohort<br><br>USA | N = 724<br><br>Lipoedema group n = 93<br>Age in years: mean ± sd 57.0 ± 13.0<br>F/M (%): 100.0% / 0.0%<br><br>Control group 1 (healthy) n = 106<br>Age in years: mean ± sd 53.0 ± 13.0<br>F/M (%): 93.0% / 7.0%<br><br>Control group 2 (Lymphoedema) n = 407<br>Age in years: mean ± sd 62.0 ± 15.0<br>F/M (%): 79.0% / 21.0%<br><br>Control group 3 (Lymphovascular disease) n = 118<br>Age in years: mean ± sd 45.0 ± 23.0<br>F/M (%): 63.0% / 27.0% | B530 | BMI (kg/m²): mean ± sd<br>38.0 ± 9.0 | BMI (kg/m²): mean ± sd CG 1 / CG 2 / CG 3<br>28.0 ± 7.0 / 31.0 ± 10.0 / 30.0 ± 10.0 |
| | | P | Comorbidities: proportions<br>Obesity: 18.93<br>Hypertension: 4.35<br>Hypercholesterolemia: 4.86<br>Chronic venous insufficiency: 3.84<br>Breast cancer: 1.02<br>Cellulitis: 1.79<br>Chronic back pain: 5.37<br>General anxiety disorder: 3.58<br>Osteoarthritis: 5.88<br>Hypothyroidism: 5.88<br>Major depressive disorders: 4.86<br>Diabetic mellitus: 1.53<br>Insomnia: 1.79<br>Asthma: 4.35<br>Migraine: 3.84<br>Melanoma: 0.00<br>Uterine fibroid: 1.02<br>Abnormal uterine bleeding: 2.05<br>Endometriosis: 1.28 | Comorbidities: proportions CG 1 / CG 2 / CG 3:<br>Obesity: 8.61 / 9.42 / 12.58<br>Hypertension: 7.09 / 8.13 / 7.23<br>Hypercholesterolemia 4.81 / 6.30 / 4.40<br>Chronic venous insufficiency: 1.77 / 6.08 / 2.83<br>Breast cancer: 16.96 / 5.97/ 1.26<br>Cellulitis: 2.78 / 5.33 / 6.60<br>Chronic back pain: 8.61 / 5.22 / 3.77<br>General anxiety disorder: 9.37 / 4.84* / 5.35<br>Osteoarthritis: 5.06 / 4.79 / 3.46<br>Hypothyroidism: 4.56 / 4.47 / 3.14<br>Major depressive disorders: 5.82 / 3.98 / 2.83<br>Diabetic mellitus: 2.28 / 3.12 / 1.57<br>Insomnia: 5.82 / 2.69 / 3.46<br>Asthma: 4.05 / 2.53 / 1.89<br>Migraine: 3.04 / 1.45 / 1.57<br>Melanoma: 0.51 / 1.29 / 0.00<br>Uterine fibroid: 3.04 / 1.08 / 0.31<br>Abnormal uterine bleeding: 2.78 / 0.75 /0.31<br>Endometriosis: 3.04 / 0.65 / 0.31 |
| | | P | Race: % of population<br>White: 51.0%<br>Black: 1.0%<br>Hispanic: 9.0%<br>Asian: 2.0%<br>Other: 37.0% | Race: % of population CG 1/ CG 2/ CG 3<br>White: 52.0% / 53.0% / 52.0%<br>Black 2.0% / 3.0% / 3.0%<br>Hispanic 9.0% / 6.0% / 5.0%<br>Asian 17.0% / 9.0% / 3.0%<br>Other 20.0% / 29.0% / 37.0% |
| Schlossen-hauer et al., 2021<br><br>Cohort<br><br>Germany | N = 69<br><br>Age in years: mean ± sd 50.6 ± 12.8<br><br>Lipoedema stage: % of population<br>III: 100.0% | B530 | BMI (kg/m²) (n = 56): mean ± sd 33.4 ± 7.1 | n/a |
| | | P | FLQA-lk (0-5) (n = 20): mean ± sd<br>Physical complaints 4.2 ± 0.4<br>Daily life 4.2 ± 2.8<br>Social life 4.3 ± 0.4<br>Emotional well-being 4.1 ± 0.5<br>Therapy of lymphatic disorder (n = 13) 4.2 ± 0.5<br>Global score 4.2 ± 0.4 | |
| | | P | Quality of life VAS (0-10) (n = 20): mean ± sd 3.7 ± 1.4 | |

| | | | | |
|---|---|---|---|---|
| | | P | General health status VAS (0-10) (n=20): mean ± sd 4.4 ± 1.7<br><br>Lymphatic disease health status VAS (0-10) (n=20): mean ± sd 3.5 ± 1.4 | |
| | | P | | |
| Schneider, 2018<br><br>Randomised clinical trial<br><br>Germany | N = 30<br><br>Lipoedema group 1 n = 15<br>Lipoedema group 2 n = 15<br><br>Age in years (n = 30): mean (range) 53.2 (23-75)<br><br>Duration of disease in years (n = 30): mean ± sd<br>12.5 ±9.1<br><br>Lipoedema stage (n = 30): % of population<br>I: 90.0%<br>II: 10.0% | B280<br><br>B530<br><br>B710<br><br>S810<br><br>P<br><br><br><br><br><br><br>E580<br>E580 | Pain in limbs: % of population 73.0%<br><br>BMI (kg/m²): mean 35.6<br><br>Impaired limb flexibility: % of population 63.0%<br><br>Skin alterations: % of population 40.0%<br><br>Quality of life with PLC: mean ± sd LG 1/ LG 2<br>Physical performance (0-32): 15 ± 6.6 / 16.9 ± 4.4<br>Ability to relax (0-32): 17.3 ± 5.0 / 19.7 ± 3.3<br>Positive mood (0-20): 8.7 ± 4.0 / 10.6 ± 3.0<br>Negative mood (0-32): 19.6 ± 6.3 / 21.6 ± 4.0<br><br>Treatment: % of population<br>Applied compression bandaging on a daily basis: 53.3%<br>Under regular medical treatment: 56.7% | n/a |
| Sørlie et al., 2022<br><br>Clinical trial<br><br>Norway | N = 9<br><br>Age in years: mean ± sd 46.9 ± 9.0 | B280<br><br>B530<br><br>B530<br><br>B530<br><br><br><br><br><br>B545<br><br><br>B545<br>B545<br>B598 | Pain VAS (0-10): mean ± sem 4.6 ± 0.69<br><br>BMI (kg/m²): mean ± sem 36.7 ± 4.5<br><br>Waist-to-hip ratio: mean ± sem 0.8 ± 0.02<br><br>Body composition: mean ± sem<br>*Method: Bioelectrical impedance analysis*<br>Fat mass (%): 46.5 ± 1.4<br>Fat mass (kg): 47.8 ± 3.1<br>Fat free mass (kg): 54.3 ± 1.7<br>Skeletal muscle mass (kg): 30.1 ± 1.0<br>Body water (l): 41.0 ± 1.4<br><br>Laboratory test results: mean ± sem<br>Natrium (mmol/L): 140.3 ± 0.5<br>Potassium (mmol/L): 4.1 ± 0.07<br>Glucose (mmol/L): 3.2 ± 0.3 | |

| | | | | |
|---|---|---|---|---|
| | | B598 | HbA1c (mmol/mol): 34.6 ± 1.1 | |
| | | D230 | Physical activity level: mean ± sem<br>1.4 ± 0.02 | |
| | | D230 | Steps per day: mean ± sem<br>5241.7 ± 465.8 | |
| | | P | Norwegian version of the quality of life questionnaire for lymphoedema of the leg: means ± sem<br>General life quality VAS (0-10): 5.1 ± 0.6<br>Function (0-4): 2.1 ± 14.4<br>Body image/appearance (0-4): 3.1 ± 0.2<br>Symptoms (0-4): 2.8 ± 0.2<br>Feeling (0-4): 1.9 ± 0.2<br>Total: 11.0 ± 1.05 | |
| Szolnoky et al., 2008<br><br>Controlled clinical trial<br><br>Hungary | N = 48<br><br>Lipoedema group 1 n = 21<br>Age in years: mean ± sd 52.12 ± 11.37<br><br>Lipoedema group 2 n = 17<br>Age in years: mean ± sd 56.34 ± 11.24<br><br>Control group (without Lipoedema) n = 10<br>Age in years: mean ± sd 42.31 ± 8.11 | B820 | Capillary fragility number of petechiae: mean ± sd<br>*Method: Angiosterrometry*<br>LG 1: 13.95 ± 10.17<br>LG 2: 12.38 ± 9.35 | Capillary fragility number of petechiae: mean ± sd<br>*Method: Angiosterrometry*<br>4.10 ± 1.66 |
| Szolnokey et al., 2011<br><br>Controlled clinical trial<br><br>Hungary | N = 38<br><br>Lipoedema group 1 n = 19<br>Age in years: mean (range) 53.89 (31-68)<br><br>Lipoedema group 2 n = 19<br>Age in years: mean (range) 55.7 (42-80) | B280<br><br>B280 | Pain NPRS (0-10): mean ± sd<br>LG 1: 5.89 ± 1.868<br>LG 2: 5.37 ± 1.950<br><br>Pain wong-baker face: mean ± sd<br>LG 1: 5.26 ± 2.423<br>LG 2: 5.16 ± 2.340 | n/a |
| Szolnoky et al., 2012<br><br>Cross-sectional study<br><br>Hungary | N = 28<br><br>Lipoedema group n = 14<br>Age in years: mean ± sd 40.3 ± 9.0<br><br>Control group (without lipoedema) n = 14<br>Age in years: mean ± sd 36.0 ± 7 | B410<br><br>S410 | Left ventricular (LV) functional parameter (%): mean ± sd<br>*Method: Two-dimensional echocardiography*<br>LV ejection fraction: 67.4 ± 5.9<br><br>LV and aortic anatomical parameters: mean ± sd<br>*Method: Two-dimensional echocardiography*<br>LV end-diastolic diameter (mm): 49.4 ± 3.4<br>LV end-systolic diameter (mm): 30.8 ± 2.8 | Left ventricular (LV) functional parameter (%): mean ± sd<br>*Method: Two-dimensional echocardiography*<br>LV ejection fraction: 68.1 ± 5.4<br><br>LV and aortic anatomical parameters: mean ± sd<br>*Method: Two-dimensional echocardiography*<br>LV end-diastolic diameter (mm): 48.4 ± 3.0<br>LV end-systolic diameter (mm): 30.1 ± 3.5 |

| | | | | |
|---|---|---|---|---|
| | | | Interventricular septum (mm): 9.4 ± 1.1<br>LV posterior wall (mm): 9.0 ± 1.0<br>Ascending aortic systolic diameter (mm): 30.0 ± 3.2<br>Ascending aortic diastolic diameter (mm): 27.8 ± 3.3<br>Aortic diameter change (mm): 2.20 ± 0.92<br>Aortic strain: 0.082 ± 0.040<br>Aortic distensibility ($cm^2$/dynes 10-6): 2.24 ± 1.07<br>Aortic stiffness index (ß): 9.5 ± 7.45 | Interventricular septum (mm): 9.4 ± 0.8<br>LV posterior wall (mm): 9.1 ± 1.0<br>Ascending aortic systolic diameter (mm): 25.5 ± 3.6<br>Ascending aortic diastolic diameter (mm): 22.3 ± 3.1<br>Aortic diameter change (mm): 3.21 ± 0.87<br>Aortic strain: 0.143 ± 0.038<br>Aortic distensibility ($cm^2$/dynes 10-6): 4.38 ± 1.61<br>Aortic stiffness index (ß): 3.76 ± 1.22 |
| | | B420 | Blood pressure (mmHg): mean ± sd<br>Systolic: 135.4 ± 9.9<br>Diastolic: 79.1 ± 12.4 | Blood pressure (mmHg): mean ± sd<br>Systolic: 129.6 ± 13.9<br>Diastolic: 77.9 ± 8.6 |
| | | B530 | BMI ($kg/m^2$): mean ± sd 27.6 ± 1.7 | BMI ($kg/m^2$): mean ± sd 27.2 ± 3.3 |
| | | P | Comorbidities: % of population<br>Hypertension: 29.0%<br>Diabetes mellitus: 0.0%<br>Lipid metabolism disorder: 0.0%<br>Anemia: 0.0% | Comorbidities: % of population<br>Hypertension: 21.0%<br>Diabetes mellitus: 0.0%<br>Lipid metabolism disorder: 0.0%<br>Anemia: 0.0% |
| Szolnoky et al., 2017<br><br>Cross-sectional study<br><br>Hungary | N = 30<br><br>Lipoedema group n = 15<br>Age in years: median (range) 59.53 (38.0-82.0)<br><br>Control group (obese) n = 15<br>Age in years: median (range) 51.13 (33.0-64.0) | B820 | Capillary fragility number of petechiae: mean ± sd<br>*Method: Angiosterrometry*<br>Right leg: 13.38 ± 10.23<br>Left leg: 13.44 ± 11.53 | Capillary fragility number of petechiae: mean ± sd<br>*Method: Angiosterrometry*<br>Right leg: 2.47 ± 3.09<br>Left leg: 2.33 ± 2.35 |
| Tartaglione et al., 2020<br><br>Cross-sectional study<br><br>Italy | N = 54<br><br>Age in years: mean ± sd 47.1 ± 19.3 | B435 | Lymphatic function: % of population<br>*Method: Lymphoscintigraphy*<br>Collaterals and/or popliteal node uptake: 49.1%<br>Tracer stagnation areas: 14.8%<br>Dermal backflow: 2.8%<br>Deep lymphatic vessels and popliteal node uptake: 36.0%<br>Tracer appearance time inguinal lymph node <10 min: 92.5%<br>Transport index: mean (range) 9.0 (3.0–16.0) | n/a |
| | | B530 | BMI ($kg/m^2$): mean (range) 32.3 (21.7-58.5) | |

| | | | | |
|---|---|---|---|---|
| | | S420 | Lymphatic anatomy: % of population<br>*Method: Lymphoscintigraphy*<br>Normal visualization lymphatic vessel: 33.3%<br>Complete visualization lymphatic pathways: 92.5%<br>Tortuous course lymph pathway and visualization collateral flow: 75.0% | |
| Van de Pas et al., 2020<br>Cross-sectional study<br><br>The Netherlands | N = 117<br><br>Age in years: mean (range) 40.9 (21.0-64.1) | B435 | Lymphatic function: % of population<br>*Method: Lymphoscintigraphy*<br>Clearance right foot two hour post injection:<br>Disturbed (≤30%): 79.5%<br>Normal (>30%): 20.5%<br>Clearance left foot two hour post injection:<br>Disturbed (≤30%): 87.2%<br>Normal (>30%): 12.8%<br>Inguinal uptake right leg two hour post injection:<br>Disturbed (≤10%): 60.3%<br>Normal (>10%): 39.7%<br>Inguinal uptake left leg two hour post injection:<br>Abnormal (≤10%): 64.7%<br>Normal (>10%): 35.3% | n/a |
| Van Esch-Smeenge et al., 2017<br><br>Cross-sectional study<br><br>The Netherlands | N = 44<br><br>Lipoedema group n = 22<br>Age in years: mean ± sd 39.23 ± 13.0<br><br>Control group (Obesity) n = 22<br>Age in years: mean ± sd 48.45 ± 9.9 | B455<br><br>B530<br><br>B730 | 6MWT (m): mean ± sd<br>494.1 ± 116.0<br><br>BMI (kg/m²): mean ± sd 33.59 ± 8.3<br><br>Muscle strength quadriceps (newton): mean ± sd<br>*Method: MicroFET*<br>Right: 269.7 ± 67.8<br>Left: 259.9 ± 77.3 | 6MWT (m): mean ± sd<br>523.9 ± 62.69<br><br>BMI (kg/m²): mean ± sd 35.06 ± 4.3<br><br>Muscle strength quadriceps (newton): mean ± sd<br>*Method: MicroFET*<br>Right: 400.3 ± 69.1<br>Left: 401.5 ± 75.9 |
| Witte et al., 2020<br><br>Prospective cohort<br><br>Germany | N = 130<br><br>Age in years: median 35<br><br>Lipoedema stage: % of population<br>I: 29.0%<br>II: 71.0%<br><br>Lipoedema location: % of population<br>Both arms and legs: 75.0%<br>Only legs: 25.0% | B1801<br><br>B2702<br><br>B280<br><br>B435<br><br>B530<br><br>B780 | Aesthetic impairment VAS (0-10): mean ± sd 8.71 ± 2.26<br><br>Sensitivity to touch VAS (0-10): mean ± sd 7.14 ± 1.9<br><br>Pain VAS (0-10): mean ± sd 6.47 ± 2.05<br><br>Swelling VAS (0-10): mean ± sd 6.75 ± 2.41<br><br>BMI (kg/m²): mean ± sd 28.4 ± 4.5<br><br>Feeling tension VAS (0-10): mean ± sd 7.56 ± 1.72 | n/a |

| | | | | |
|---|---|---|---|---|
| | | B780 | Feeling of heavy legs VAS (0-10): mean ± sd 8.42 ± 1.80 | |
| | | B820 | Bruising VAS (0-10): mean ± sd 7.18 ± 1.93 | |
| | | B840 | Itching VAS (0-10): mean ± sd 4.0 ± 3.3 | |
| | | D455 | Running impairment VAS (0-10): mean ± sd 5.28 ± 3.04 | |
| | | P | Occupational impairment VAS (0-10): mean ± sd 4.97 ± 2.63 | |
| | | P | General impairment VAS (0-10): mean ± sd 7.79 ± 2.11 | |
| | | P | Varicose veins: % of population 35.0% | |
| | | <br>E580<br>E580 | Treatment: % of population<br>Manual lymphatic drainage therapy: 88.9%<br>Compression garments: 95.2% | |
| Wold et al., 1951<br><br>Case series<br><br>USA | N = 119<br><br>F/M (%) = 99.2%/ 0.8%<br><br>Positive family history: % of population 16.0%<br><br>Age onset in years: % of population<br>0 - 8: 6.7%<br>11 - 20: 13.4%<br>21 – 30: 16.0%<br>31 – 40: 19.3%<br>41 – 50: 21.8%<br>51 – 60: 10.1%<br>61 – 70: 5.0%<br>71 – 80: 0.8%<br>Unknown: 6.7% | B180<br><br>B435<br><br>B28015<br><br>B540<br><br><br><br><br>P | Neurosis about legs appearance: % of population 29.0%<br><br>Pitting edema: % of population 24.0%<br><br>Pain in lower extremities: % of population 40.0%<br><br>Basal metabolic rate in percentage (n = 30): % of population<br>*Method: unknown*<br>Less than 0%: 90.0%<br>Elevated: 10.0%<br><br>Race: % of population<br>American: 46.2%<br>British Isles: 13.4%<br>Jewish: 13.4%<br>North Europe: 12.6%<br>Scandinavia: 5.9%<br>Canadian: 3.4%<br>East Europe: 2.5%<br>South Europe: 1.7%<br>African: 0.8% | n/a |
| Wollina et al., 2019 | N = 111 | B280 | Pain VAS (0-10): median ± sd 7.8 ± 2.1 | n/a |

| | | | |
|---|---|---|---|
| Retrospective cohort<br><br>Germany | Age in years: median ± sd 44.0 ± 16.8<br>F/M (%): 99.2% / 0.8%<br><br>Lipoedema stage: % of population<br>I: 6.3%<br>II: 45.5%<br>III: 43.2%<br><br>Lipoedema location: % of population<br>Involvement legs: 100.0%<br>Involvement arms: 24.0%<br>Dominant involvement upper legs: 97.3%<br>Dominant involvement lower legs: 1.8% | P | Comorbidities (%):<br>Hypertension: 27.3%<br>Obesity >35: 21.0%<br>Hypothereosis: 20.0%<br>Atopic diseases: 20.0%<br>Osteoarthritis: 19.1%<br>Lymphoedema: 10.9%<br>Varicose veins of the legs: 10.0%<br>Depression and anxiety: 6.4 |
| Wright et al., 2021<br><br>Case report<br><br>USA | N = 1<br><br>Age in years: 41.0<br><br>Age onset in years: 12.0<br><br>Lipoedema location:<br>From waist to ankle, arms affected. | B420<br><br><br>B430<br>B430<br>B430<br>B430<br>B430<br><br>B435<br><br><br><br><br>B530<br><br><br>B540<br>B540<br>B545<br>B545<br>B545<br>B545<br>B545<br>B598<br>B610<br>B610 | Blood pressure (mmHg) (systolic/ diastolic): 110/58<br><br>Laboratory test results<br>White blood cell (K/μL): 3.0<br>Hemoglobin (g/dL): 9.8<br>Hematocrit (%): 33.0%<br>Platelet count (K/μL): 380<br>Absolute neutrophils (cells/μL ): 1700<br><br>Lymphathic function<br>*Method: Lymfoscintigram*<br>Radiotracer uptake in lymph nodes with slightly delayed clearance in all 4 limbs; interpreted as normal.<br><br>BMI (kg/m²): 38<br><br>Laboratory test results:<br>Carbon dioxide (mmol/L): 22<br>Total protein (g/dL): 5.5<br>Albumin (g/dL): 2.0<br>Sodium (mmol/L): 137<br>Potassium (mmol/L): 3.7<br>Calcium (mg/dl): 8.4<br>Chloride (mmol/L): 100<br>Glucose (mg/dL ): 87<br>Creatinine (mg/dL): 0.6<br>Blood urea nitrogen (mg/dL): 10 |

| | | | | |
|---|---|---|---|---|
| Ziegler et al., 2020<br><br>Case report<br><br>Germany | N = 1<br><br>Age in years: 37.0 | B530 | BMI (kg/m²): 62.4 | |

a: late transmitral flow velocity, AS: area strain, B: body function, BMI: body mass index, CDT: combined decongestive therapy, CG: control group, CI: confidence interval, cm: centimeter, CS: circumferential strain, D: activities and participation, dL: deciliter, E: environmental factor, e: early transmitral flow velocity, EQ-5D: EuroQol 5D, F: female, FLQA-lk: The Freiburg Quality of Life Assessment for lymphatic disorders, Short Version, g: gram, Hb: haemoglobin, HbA1C: hemoglobin A1C, HOMA-IR: Homeostatic Model Assessment for Insulin Resistance, IPAQ: International Physical Activity Questionnaire, IQR: interquartile range, kcal: kilocalorie, kg: kilogram, K/µL: thousand cells of microliter, L: liter, LG: lipoedema group, LS: longitudinal strain, LV: left ventricle, m: meter, M: male, max.: maximum, MET: Metabolic Equivalent of Task, mg: microgram, µg: microgram, min: minute, ml: milliliter, MLD: manual lymphatic drainage, mm: millimeter, µm: micrometer, mmHg: millimetres of mercury, mmol: millimole, N: number, n/a: not applicable, ng: nanogram, P: personal factor, PLC: Profile of Quality of Life in the Chronically Ill, RS: radial strain, R0 and R00(Ω): impedance velues inversely related to extracellular and total tissue water volume, S: body structure, se: standard error, sem: standard error of mean, SF-36: Short Form 36, sd: standard deviation, SQS: Sleep Quality Scale, UK: United Kingdom, USA: United States of America, VAS: Visual Analogue Scale, WHOQOL-BREF: World Health Organization Quality of Life Questionnaire - BREF, WOMAC: Western Ontario and McMaster Universities Arthritis Index, 3D: three-dimensional, 3DS: three-dimensional strain, 6MWT: six-minute walk test.

## Supplementary Table S3. Study characteristics gray literature

| **Author(s), year, design, Country** | **Population and lipoedema characteristics** | **Outcomes** | | |
|---|---|---|---|---|
| | | **ICF** | **Lipoedema group(s)** | **Control group(s)** |
| Grigoriadis et al., 2021<br><br>Cross-sectional study<br><br>UK | N = 200<br><br>Age in years: mean ± sd 47.0 ± 13.5<br><br>Age at onset in years: mean ± sd 16.8 ± 9.0<br><br>Duration of disease in years: mean ± sd 29.2 ± 12.9<br><br>Positive family history: % of population 58.2% | B270<br><br>B435<br><br>B435<br><br>B435<br><br>B530<br><br>B530<br><br>B530<br><br><br><br><br>B820 | Tender to touch and pain: % of population 71.0%<br><br>Oedema of ankle: % of population 51.1 %<br><br>Oedema of leg/lower limb: % of population 38.3%<br><br>Intermittent oedema: % of population 10.6%<br><br>BMI (kg/m²): mean ± sd 33.4 ± 7.2<br><br>Waist-to-hip ratio: mean ± sd 0.76 ± 0.07<br><br>Responsiveness to dieting: % of population<br>Disproportional response: 86.7 %<br>No loss: 7.8 %<br>Equal loss all over: 5.4 %<br><br>Bruise easily: % of population 90.3% | n/a |

| | | | | |
|---|---|---|---|---|
| | | P | Race: % of population<br>White British: 92.5 % | |
| | | P | SF-36 (n = 135): mean ± sd<br>Physical functioning: 61.1 ± 28.0<br>Role limitations physical: 58.9 ± 42.9<br>Role limitations emotional: 57.9 ± 42.9<br>Vitality: 40.2 ± 23.9<br>Emotional/mental well-being: 60.1 ± 19.9<br>Social functioning: 64.7 ± 27.1<br>Bodily pain: 57.1 ± 27.1<br>General health: 49.5 ± 21.2 | |
| | | P | Comorbidities: % op population<br>Pes planus: 22.2%<br>Venous problems and lymphoedema: 13.2%<br>Hyper mobility: 17.8 %<br>Varicose veins with symptoms: 2.6%<br>Uncomplicated varicose veins: 25.3%<br>Mild superficial venous problems: 19.5%<br>No visible or palpable varicose veins: 52.6% | |
| Schwarze, 2018<br><br>Randomised controlled trial<br><br>Germany | N = 38<br><br>Lipoedema group 1: n = 21<br>Lipoedema group 2: n = 17<br><br>Age in years (n = 38): mean ± sd 45.8 ± 10.3<br><br>Positive family history: % of population 68.4% | B270 | Dolorimeter (kg/cm$^2$): mean ± sd<br>LG 1: 2.64 ± 0.93<br>LG 2: 2.44 ± 0.88 | n/a |
| | | B280 | Pain VAS (0-10): mean ± sd<br>LG 1: 3.4 ± 2.4<br>LG 2: 3.9 ± 2.8 | |
| | | B455 | 6MWT (m): (mean ± sd)<br>LG 1: 596.2 ± 79.1<br>LG 2: 520.7 ± 144.7 | |
| | | B530 | BMI (kg/m$^2$): mean ± sd 31.0 ± 7.0 | |
| | | B530 | Body composition: mean ± sd<br>*Method: Dual-energy X-ray absorptiometry*<br>Fat mass index: 14.8 ± 5.1<br>Fat mass legs: | |

| | |
|---|---|
| | LG 1 (kg): 18.89 ± 7.94<br>LG 2 (kg): 17.80 ± 5.99<br>Fat free mass legs:<br>LG 1 (kg): 15.75 ± 2.84<br>LG 2 (kg): 14.13 ± 2.32 |
| B820 | Bruising VAS (0-10): mean ± sd<br>LG 1: 6.4 ± 2.9<br>LG 2: 7.3 ± 2.0 |
| B435 | Swelling legs: VAS (0-10): mean ± sd<br>Lipoedema group 1: 5.3 ± 2.0<br>Lipoedema group 2: 5.8 ± 2.7 |
| B435 | Swelling legs: % of population<br>At night: 81.6%<br>Warm temperatures: 78.9%<br>During sitting: 55.3%<br>During standing: 42.0% |
| D450 | Mobility (walking distance, endurance) VAS (0-10): mean ± sd<br>LG 1: 5.6 ± 2.7<br>LG 2: 5.3 ± 2.7 |
| D920 | Sports activities (times/week): % of population LG 1/ LG 2<br>0: 30.0% / 44.0%<br>1- 2: 15.0% / 6.0%<br>2-3: 35.0% / 13.0%<br>3-4: 10.0% / 25.0%<br>5: 10.0% / 13.0% |
| D920 | Mean exercise time (minutes/activity): % of population LG 1/ LG 2<br><30: 7.0% / 0.0%<br>30: 14.0% / 0.0%<br>30 -60: 36.0% / 33.0%<br>60-120: 21.0% / 56.0%<br>120-180: 21.0% / 11.0% |

| | | |
|---|---|---|
| | E580 | Treatment: % of population<br>Lymph drainage treatment: 45.0%<br>Compression stockings:<br>Every day: 45.0%<br>3-4 times a week: 19.0%<br>1-2 times a week: 8.0%<br>Do not wear: 8.0%<br>Do not have any: 10.8% |

B: body function, BMI: body mass index, cm: centimeter, D: activities and participation, E: environmental factor, kg: kilogram, LG: Lipoedema group, m: meter, N: number, n/a: not applicable, P: personal factor, S: body structure, sd: standard deviation, SF-36: Short Form 36, UK: United Kingdom, VAS: Visual Analogue Scale, 6MWT: six-minute walk test.
