# Supplementary material for: Functioning of People with Lipoedema According to All Domains of the International Classification of Functioning, Disability and Health: A Scoping Review
Source: Int J Environ Res Public Health. 2023 Jan 21;20(3):1989. doi: 10.3390/ijerph20031989 (PMC9915552; doi:10.3390/ijerph20031989)
Supplement: Supplementary file 1 [file ijerph-20-01989-s001.zip › Supplementary files C L.M. Kloosterman.pdf]

Supplementary File S1. Detailed information on the method of critical appraisal of individual sources of evidence

The methodological quality was assessed using critical assessment instruments. Two researchers (L.M.K. and A.H.) separately assessed the quality of the included studies. Conflicting assessments were discussed until consensus was reached. If no consensus was reached, a meeting with a third researcher (R.D.) took place. Prior to the quality assessment, the process was tested by the researchers using a series of previously excluded references.

The quantitative publications were assessed using the Effective Public Health Practice Project (EPHPP) instrument [1]. The EPHPP instrument was chosen because it can be used to assess the methodological quality of a range of study types, it was validated for use in public health research and it has fair inter-rater reliability for the individual domain scores and excellent agreement for the final grade [1,2]. The overall assessment was scored in six areas, i.e., selection bias, study design, confounders, blinding, data collection methods, withdrawals, and drop-outs. Items were scored with "weak", "moderate", "strong", "cannot tell", or in some cases "not applicable". The assessors were instructed not to include an item in the total score if it was scored as "not applicable". Studies were rated as strong if none of the domains scored weak individually, with at least four domains rated as strong. Studies with fewer than four strong ratings or with one weak rating were rated as moderate. Studies with two or more weak ratings were assessed as weak.

The qualitative publications were assessed using the Critical Appraisal Skills Program (CASP) instrument [3]. The ten questions in this instrument are divided into three sections (A. Are the results valid, B. What are the results, C. Will the results help locally/ how valuable is the research) and are scored with "yes", "no", or "can't tell". The Authority, Accuracy, Coverage, Objectivity, Date and Significance (AACODS) checklist was used to assess the included gray literature on trustworthiness and relevance [4]. All items were scored with "yes", "no", or "do not know". To the authors' knowledge, no information is available on the validity or reliability of the CASP and the AACODS. Cohen's Kappa was used to calculate the agreement between the researchers' assessments. The critical appraisal was not used in data synthesis or to exclude publications.

References

1. Thomas, B.H.; Ciliska, D.; Dobbins, M.; Micucci, S. A Process for Systematically Reviewing the Literature: Providing the Research Evidence for Public Health Nursing Interventions. *Worldviews on Evidence-Based Nursing* **2004**, *1*, 176–184, doi:10.1111/j.1524-475X.2004.04006.x.
2. Armijo-Olivo, S.; Stiles, C.R.; Hagen, N.A.; Biondo, P.D.; Cummings, G.G. Assessment of Study Quality for Systematic Reviews: A Comparison of the Cochrane Collaboration Risk of Bias Tool and the Effective Public Health Practice Project Quality Assessment Tool: Methodological Research: Quality Assessment for Systematic Reviews. *Journal of Evaluation in Clinical Practice* **2012**, *18*, 12–18, doi:10.1111/j.1365-2753.2010.01516.x.
3. CASP Qualitative Checklist Critical Appraisal Skills Programme Available online: https://casp-uk.net/images/checklist/documents/CASP-Qualitative-Studies-Checklist/CASP-Qualitative-Checklist-2018_fillable_form.pdf (accessed on 4 January 2022).
4. Tyndall, J. AACODS Checklist Available online: http://dspace.flinders.edu.au/dspace/ (accessed on 4 January 2022).
