# Supplementary material for: Functioning of People with Lipoedema According to All Domains of the International Classification of Functioning, Disability and Health: A Scoping Review
Source: Int J Environ Res Public Health. 2023 Jan 21;20(3):1989. doi: 10.3390/ijerph20031989 (PMC9915552; doi:10.3390/ijerph20031989)
Supplement: Supplementary file 1 [file ijerph-20-01989-s001.zip › Supplementary files D L.M. Kloosterman .pdf]

Supplementary Table S4. Methodological quality assessment using the Effective Public Health Practice Project (EPHPP) instrument

Study	Selection Bias	Study Design	Confounders	Blinding	Data collection methods	Withdrawals/ Dropouts	Global rating*
Amann-Vesti et al., 2001	W	W	W	W	S	N/A	W
Amato et al., 2020	W	W	S	W	W	N/A	W
Amato et al., 2021	W	W	S	M	S	N/A	W
Angst et al., 2021	W	W	W	W	S	N/A	W
Atan et al., 2020	W	S	S	M	W	S	W
Beltran et al., 2016	N/A	W	M	W	W	N/A	W
Buso et al., 2022	W	W	N/A	M	W	N/A	W
Cannataro, 2021	W	W	N/A	W	W	N/A	W
Cellina et al., 2020	N/A	W	S	W	S	N/A	W
Crescenzi et al., 2019	W	W	S	W	S	N/A	W
Dadras et al., 2017	W	M	N/A	W	W	M	W
Dietzel et al., 2015	W	W	S	W	S	N/A	W
Di Renzo et al., 2021	M	S	S	W	S	S	M
Erbacher et al., 2020	W	W	N/A	W	W	S	W
Fink et al., 2020	N/A	M	S	W	W	W	W
Forner-Cordero et al., 2021	W	M	N/A	W	S	S	W
Gensior et al., 2020	W	W	N/A	W	W	N/A	W
Ghods et al., 2020	M	M	N/A	W	W	N/A	W
Gould et al., 2019	W	W	N/A	M	S	N/A	W
Hamatschek et al., 2022	M	W	N/A	W	S	N/A	W
Harwood et al., 1996	W	W	W	W	S	N/A	W
Herbst et al., 2015	N/A	W	N/A	W	W	N/A	W
Hirsch et al., 2018	W	W	W	M	W	N/A	W
Iker et al., 2018	W	W	W	W	W	N/A	W
Jeziorek et al., 2022	W	S	S	W	S	S	W
Kruppa et al., 2020	N/A	M	S	W	W	M	W
Lohrman et al., 2009	W	W	N/A	W	S	N/A	W
Marshall et al., 2011	W	W	W	W	W	N/A	W
Münch, 2017	W	M	N/A	W	W	W	W
Nemes et al., 2018	W	W	W	W	W	N/A	W

Nemes et al., 2019	W	W	S	W	W	S	W
Nemes et al., 2020	W	W	S	W	S	S	W
Rapprich et al., 2011	M	M	N/A	W	W	W	W
Rasmussen et al., 2022	M	W	N/A	W	W	N/A	W
Rockson et al., 2022	N/A	M	S	W	S	N/A	M
Schlossenhauer et al., 2021	W	M	N/A	W	M	S	W
Schneider, 2018	M	S	W	M	S	S	M
Sørlie et al., 2022	W	M	N/A	W	S	S	W
Szolnoky et al., 2008	W	S	W	W	W	S	W
Szolnoky et al., 2011	W	S	W	W	W	W	W
Szolnoky et al., 2012	W	W	S	W	W	N/A	W
Szolnoky et al., 2017	W	W	S	W	W	N/A	W
Tartaglione et al., 2020	W	W	N/A	W	W	N/A	W
Van de Pas et al., 2020	W	W	N/A	W	S	W	W
Van Esch-Smeenge et al., 2017	W	W	S	W	S	N/A	W
Witte et al., 2020	M	M	N/A	W	S	W	W
Wold et al., 1951	W	W	N/A	W	W	N/A	W
Wollina et al., 2019	W	M	N/A	W	S	W	W
Wright et al., 2021	N/A	W	N/A	W	W	N/A	W
Ziegler et al., 2020	W	W	N/A	W	S	N/A	W

M: moderate, N/A: not applicable, S: strong, W: weak.

* Studies were rated as strong if there was no individual weak score in any of the domains, with at least four domains rated as strong. Studies with fewer than four strong ratings or with one weak rating were rated as moderate. Studies with two or more weak ratings were assessed as weak.

Supplementary Table S5. Methodological quality assessment using the Critical Appraisal Skills Program (CASP) instrument

Items	Study
	Melander et al., 2021
Section A: Are the results valid?	
1. Was there a clear statement of the aims of the research?	Yes
2. Is a qualitative methodology appropriate?	Yes
3. Was the research design appropriate to address the aims of the research?	Can't tell
4. Was the recruitment strategy appropriate to the aims of the research?	Yes
5. Was the data collected in a way that addressed the research issue?	Yes
6. Has the relationship between researcher and participants been adequately considered?	Can't tell
Section B: What are the results?	
7. Have ethical issues been taken into consideration?	Yes
8. Was the data analysis sufficiently rigorous?	Yes
9. Is there a clear statement of findings?	Yes
Section C: Will the results help locally?	
10. How valuable is the research?	Useful and valuable with clear implications for treatment.

Supplementary Table S6. Methodological quality assessment using The Authority, Accuracy, Coverage, Objectivity, Date and Significance (AACODS) checklist

Items	Study	
	Grigoriadis et al., 2021	Schwarze et al., 2021
Authority		
Individual author:		
Associated with a reputable organisation?	Yes	Yes
Professional qualifications or considerable experience?	Yes	?
Produced/published other work (grey/black) in the field?	Yes	No
Recognised expert, identified in other sources?	Yes	No
Cited by others? (use Google Scholar as a quick check)	Yes	Yes
Higher degree student under "expert" supervision?	No	Yes
Organisation or group:		
Is the organisation reputable? (e.g. W.H.O)	Yes	Yes
Is the organisation an authority in the field?	Yes	?
In all cases:		
Does the item have a detailed reference list or bibliography?	Yes	Yes
Accuracy		
Does the item have a clearly stated aim or brief?	Yes	Yes
Is so, is this met?	Yes	Yes
Does it have a stated methodology?	Yes	Yes
If so, is it adhered to?	Yes	Yes

Has it been peer-reviewed?	No	Yes
Has it been edited by a reputable authority?	?	?
Supported by authoritative, documented references or credible sources?	Yes	Yes
Is it representative of work in the field?	Yes	Yes
If No, is it a valid counterbalance?	n/a	n/a
Is any data collection explicit and appropriate for the research?	Yes	Yes
If item is secondary material (e.g. a policy brief of a technical report) refer to the original.	n/a	n/a
Is it an accurate, unbiased interpretation or analysis?	Yes	Yes
Coverage		
Are any limits clearly stated?	Yes	Yes
Objectivity		
Opinion, expert or otherwise, is still opinion: is the author's standpoint clear?	Yes	Yes
Does the work seem to be balanced in presentation?	Yes	Yes
Date		
Does the item have a clearly stated date related to content?	Yes	Yes
No easily discernible date is a strong concern.		
If no date is given, but can be closely ascertained, is there a valid reason for its absence?	n/a	n/a
Check the bibliography: have key contemporary material been included?	Yes	Yes
Significance		
Is the item meaningful? (this incorporates feasibility, utility and relevance)	Yes	Yes
Does it add context?	Yes	Yes
Does it enrich or add something unique to the research?	Yes	Yes
Does it strengthen or refute a current position?	Yes	Yes
Would the research area be lesser without it?	Yes	Yes
Is it integral, representative, typical?	Yes	Yes
Does it have impact? (in the sense of influencing the work or behaviour of others)	Yes	Yes

n/a: not applicable
